# From Molecules to Metabolomes, Understanding Symbiosis through Small Molecules

**DOI:** 10.1021/acs.jnatprod.5c01360

**Published:** 2026-03-18

**Authors:** Cristina Bez, Yasin El Abiead, Andrés M. Caraballo-Rodríguez

**Affiliations:** † Skaggs School of Pharmacy and Pharmaceutical Sciences, 8784 University of California San Diego, 9500 Gilman Drive, San Diego, California 92093-0751, United States; ‡ Bacteriology Group, International Centre for Genetic Engineering and Biotechnology (ICGEB), Padriciano 99, Trieste 34149, Italy; § Institute of Analytical Chemistry, Department of Chemistry, University of Natural Resources and Life Sciences, Vienna, Vienna 1190, Austria

## Abstract

Symbiosis, from Greek “living together” refers to the close association among organisms. Although these associations are found everywhere in nature, we do not know how these relationships are established or maintained over time. In this Perspective, we will focus on interorganism interactions involving microbes and eukaryotic hosts, particularly animals, plants, and humans, where symbiosis plays a critical role in health, development, and ecological fitness. We will focus on the chemical crosstalk between host and symbiont mediated by specialized small molecules. Finally, we suggest some steps for applying mass spectrometry-based metabolomic approaches to accelerate the understanding of these complex interactions.

## Graphical abstract

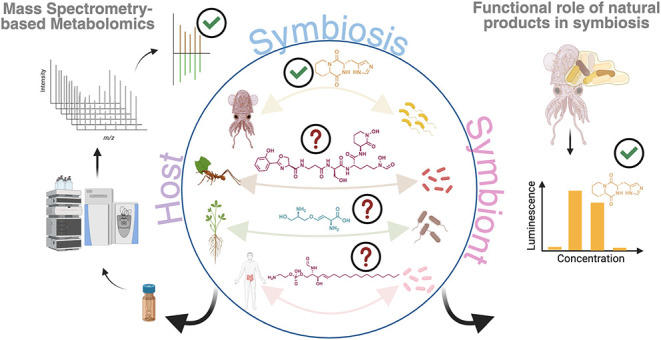

## Introduction

Among the totality of biological partnerships, **symbiosis** stands out as one of the most intimate and evolutionarily long-term forms of interaction between two or more organisms belonging to different species. This definition excludes organisms that interact casually or merely co-occur in the same environment, but it does not assess the nature of the association, which can span a wide range of ecological outcomes including mutualism, commensalism, and parasitism, and may be obligate or facultative.^1^ Such interactions are widespread across all domains of life and are often deeply integrated into host physiology and development. In this context, we focus on microbe–eukaryote symbioses, particularly those involving animals, plants, and humans, where communication and coordination depend on complex chemical exchanges mediated by specialized small molecules known as natural products.^2^ We will also follow convention by calling the microbial participant the symbiont and the eukaryotic partner the host.

Over time, selective pressures have shaped symbiotic relationships in which one partner (most often a bacterium or fungus) produces bioactive small molecules to communicate and influence the host’s behavior and potentially other symbionts. Relevant here is to consider that often the organisms undergoing this type of tight interaction are not only the host and a single microbe but rather a complex set of interdependent microbes (microbiome), in persistent symbiosis, whose developmental programs have coevolved.^3^ During evolution, the host’s needs and symbiont’s traits have driven partner selection and coadaptation, shaping the long-term stability of symbiotic systems. Although some associations show strong partner fidelity or even taxonomic specificity, accumulating evidence indicates that function, rather than phylogeny, is the primary determinant of symbiont choice.^4^ Depending on the system, these functionally aligned partners may be inherited maternally (vertical transmission, e.g., insect–bacteriocyte symbionts) or acquired from the environment at each generation (horizontal transmission, e.g., gut consortia).^5^ Such high-fidelity relationships exert strong evolutionary pressures that shape both symbiont and host genomes.^6^ A hallmark of this process is symbiont genome reduction, wherein only genes essential for symbiosis, such as those related to nutrient provisioning, defense, or small molecule metabolism, are retained. Mitochondria, chloroplasts and intracellular symbionts of insects, exemplify this phenomenon: originally free-living bacteria, they have become fully integrated into the host genomic and cellular context.^6–9^ There are also cases in which bacterial symbiont genes are horizontally transferred to and are actually expressed by host eukaryotic cells, which indicates a tight genetic and metabolic coupling between these symbiotic organisms.^10^ A remarkable example is *Wolbachia pipientis*, a maternally transmitted endosymbiont that infects a broad array of arthropods. Its genetic material has been identified across multiple invertebrate lineages, including fruit flies, wasps, and nematodes, highlighting its pervasive influence and evolutionary symbiotic success.^11^

Host organisms employ diverse control mechanisms to favor beneficial over detrimental symbionts. These include partner choice, which selects among available microbes, and partner manipulation, where host mechanisms actively modulate symbiont behavior and metabolism to enhance host benefit.^12^ However, a critical and still underexplored aspect of symbiosis is the initial establishment phase. It remains unclear whether partner selection is entirely host-driven, or whether symbionts also possess strategies to actively attract or manipulate host responses to ensure their selection. Despite its central importance, the **establishment** phase has received limited attention compared to **maintenance**, and few studies have systematically investigated the molecular or ecological factors that govern successful pairing between hosts and microbial partners. At the initial stage, physical and immunological host barriers play pivotal roles in shaping microbial access. In animals, barriers such as the skin or mucosal surfaces, and in plants, the root epidermis, act as gatekeepers that restrict microbial entry while still allowing chemical signaling across the interface. Both animal and plant hosts employ a diverse array of pattern recognition receptors that detect conserved microbial signatures or respond to perturbations in host cellular processes, thus enabling the host to distinguish between potential symbionts, neutral microbes, and pathogens at the earliest stages of contact.^13,14^

Beyond these structural and innate immune-based checkpoints, a key open question is whether additional chemical cues are involved in facilitating symbiotic establishment and subsequently the maintenance. Indeed, long-term, coevolved symbiotic interactions are often chemically mediated, not only through primary metabolites but also via bioactive and specialized small molecules, i.e., **natural products**, that perform signaling, defensive, or structural functions. These chemical dialogues may provide an additional layer of specificity and coordination during symbiosis, supporting compatibility and mutual benefit between host and symbiont. Natural products are now widely recognized for their precise roles in symbiotic systems, targeting defined macromolecular pathways and contributing to the regulation, stabilization, and protection of these associations.^15^ These complex molecules, derived from microbial symbionts,^15^ exhibit diverse functional roles: some deter predators of the animal host, while others shape the composition and behavior of host-associated microbial communities.^16,17^ Many of these molecules are classified as natural products primarily because they were first isolated during studies of a particular host organism, and only later found to be produced by associated bacterial symbionts. In numerous cases, the biological function of these compounds is characterized *in vitro*, and their true ecological role within the host context remains unclear as well as the molecular evidence suggesting their involvement in symbiotic relationships.

It is also important to highlight the role of small molecules as part of metabolic processes. Symbiotic systems frequently rely on microbial biotransformations and nutrient-degradation pathways that generate metabolites essential for establishing and maintaining the interaction. For instance, in plant–microbe symbioses, rhizobia^18^ reduce atmospheric nitrogen N_2_ to ammonium (NH_4_^+^), while arbuscular mycorrhizal fungi^19^ mobilize mineral nutrients, such as phosphorus, from organic complexes, directly supplying plant growth. In animal systems, termite gut microbiota^20^ degrade lignocellulose into fermentable sugars and short-chain fatty acids, and human gut bacteria (particularly Bacteroidetes and Firmicutes) convert indigestible fibers and dietary tryptophan into metabolites such as short-chain fatty acids^21,22^ (acetate, propionate, and butyrate) and indole-derivatives^23^ that modulate host immunity and physiology. In marine associations, *Vibrio fischeri* ^24^ uses host-derived chitin degradation products like disaccharide *N*,*N*′-diacetylchitobiose (chitobiose) to initiate colonization and regulate luminescence pathways. These examples illustrate how metabolites derived from nutrient breakdown are essential to symbiosis and can act as energy sources but also as signaling molecules.

In this Perspective, starting by a literature search of reported natural products involved in symbiosis, we selected a few well-established textbook examples in which natural products have been experimentally tied to symbiosis and highlight how these molecules orchestrate host–microbe interactions in plants, marine organisms, insects and human environments. By giving particular emphasis on the key challenges associated with deciphering this molecular dialogue, we also highlight the potential for discovery of natural products from metabolomes. Finally, we suggest some steps for applying mass spectrometry-based metabolomic approaches to discover natural products mediating these complex interactions.

### Natural Products in Symbiosis

By searching in literature, we found 94 molecules and some of their derivatives (Figure 1 shows 19 representative molecules) with evidence of their involvement in symbiosis (Table S1). We classified them according to their roles in either the establishment or maintenance of the interaction, based on the functions they perform and the processes they mediate. Notably, some molecules may contribute to both phases. We proposed a “***symbiosis score***” to indicate the involvement for each molecule in symbiosis according to the following evidence: **score 1**. Chemical structure has been confirmed, **score 2**. Both chemical structure and molecule’s producer have been confirmed or **score 3**. All the following, chemical structure, molecule’s producer and bioassay confirming their involvement in symbiosis have been provided.

**Figure 1:**
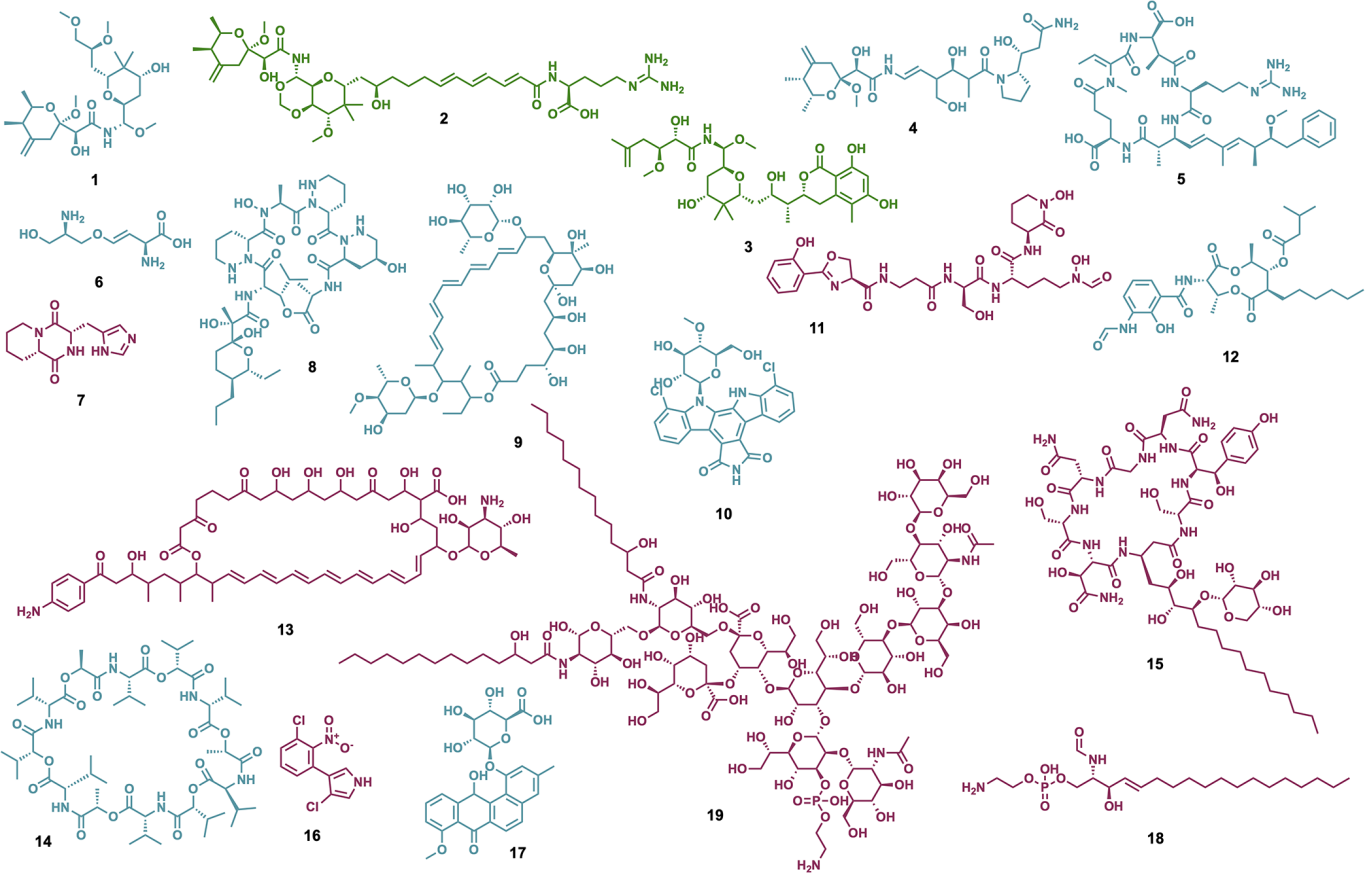
Natural products involved in symbiosis. Molecules with a ***symbiosis score*** (score 1 in dark green, score 2 in dark cyan and score 3 in dark magenta) to highlight some representative molecules reported from symbiotic systems to date: pederin (**1**), onnamide A (**2**), psymberin (**3**), nosperin (**4**), nodularin (**5**), rhizobitoxine (**6**), cyclo­(d-histidyl-l-proline) (**7**), dentigerumycin A (**8**), selvamycin (**9**), rebeccamycin (**10**), attinimicin (**11**), antimycin A1 (**12**), candicidin (**13**), valinomycin (**14**), burkholdine1213 (**15**), pyrrolnitrin (**16**), pseudonocardone A (**17**), ceramide phosphoethanolamine (**18**) and lipooligosaccharide (**19**). Additional literature-reported molecules involved in symbiosis are summarized in Table S1.

Several trends emerge from this analysis. It was evident that there has been a strong research focus on marine organisms, insects, and plants, which represent the most common host systems studied from the symbiosis perspective. A large majority of the symbiosis-involved molecules are associated with the maintenance of symbiosis, while a lower proportion are linked to its establishment (Table S1), therefore highlighting a significant knowledge gap in our molecular understanding of how symbiotic interactions are initiated. In addition, natural products linked to the initiation of symbiosis are primarily found in plant-associated systems, whereas those involved in maintenance are more broadly distributed across insects, fungi/lichens, and marine hosts.

The most represented chemical classes include alkaloids, isoflavones, and polyketide–peptide hybrids. These classes of molecules might have a specific chemical and biological versatility. Moreover, there are classes such as diketopiperazines and polyketides that showed higher average symbiosis scores, indicating stronger experimental support and validation for their roles in symbiosis. Based on the “***symbiosis score***” introduced in this perspective, only a limited number of natural products have been biologically, biochemically, and functionally validated as playing a definitive role in the establishment or maintenance of symbiosis. This likely reflects the numerous technical and biological challenges inherent to studying symbiotic interactions, as discussed in the following sections. In general, the production of complex molecules from polyketide synthase (PKS) and nonribosomal peptide synthetase (NRPS) pathways has been predominantly studied and discovered in bacterial–eukaryote symbioses from marine environments.^25^ In contrast, bacterial symbiosis with animals or plants have typically involved chemically and structurally simpler compounds such as lipids and polysaccharides. Metabolites of symbiont origin, whether confirmed or suspected, are generally structurally diverse, highlighting the remarkable chemical diversity underlying symbiotic interactions. An exception to this trend is the case of pederin (**1**), produced by bacteria living in rove beetles of the genus *Paederus*, along with its structural analogs, onnamides (**2**) and psymberin (**3**)—synthesized by sponge-associated bacteria and nosperin (**4**) produced by lichen-associated bacteria.^26^ Similar to this exception is the case of related bacteria which generate similar compounds using unrelated biosynthetic pathways, suggesting that these bacteria may have independently evolved the ability to produce distinct metabolites with specialized functions, potentially conferring adaptive advantages.^27,28^ There are also some natural products, such as nodularin (**5**),^29^ which have been found to be produced by a broader range of cyanobacteria, including both free-living and host-associated. These cases highlight the possibility that certain metabolites may serve conserved ecological functions across different microbial lifestyles or have pleiotropic function according to the conditions.

#### Leguminous Plants and Their Food Supplier Rhizobiales

Among the most extensively characterized and molecularly validated examples of plant–bacterial symbiosis are the interactions between leguminous plants and various α-proteobacteria of the order Rhizobiales.^30,31^ These microorganisms induce the formation of specialized plant structures known as root nodules, where they convert atmospheric nitrogen into ammonium in exchange for carbon and a low-oxygen environment.^32^ Symbiosis is initiated by plant-derived elicitors, including flavonoids, betaines, and aldonic acids, which act as chemoattractants and trigger the expression of symbiosis-associated genes in free-living rhizobia. These genes orchestrate the biosynthesis and secretion of lipochitooligosaccharide signals, commonly known as Nod factors, which are detected by plant lysine motif receptor-like kinases (LysM) and in turn initiate the developmental program leading to nodule organogenesis.^33^ Nod factors typically consist of a conserved β-1,4-linked oligo-*N*-acetyl-d-glucosamine backbone composed of three to six sugar units, variably modified with groups like sulfyl, acetyl, and fatty acyl chains, which dictate host specificity.^34–36^ For example, sulfylated Nod factors from *Sinorhizobium meliloti* are required for *Medicago sativa* colonization but inhibit nonhost interactions. Importantly, each rhizobial species forms a symbiotic relationship with only a specific subset of leguminous plants. This host specificity and the successful establishment of this symbiosis is partly driven by NodD-mediated activation of nod genes in response to a distinct mix of flavonoids present in the host plant’s root exudates and by the specific structure of the lipochitooligosaccharide.^30^

In the maintenance phase, other small molecules like cyclic glucans, rhizopines, and rhizobitoxine (**6**) contribute to sustaining the symbiosis. This complex chemical exchange along with the isolation of clonal populations of beneficial nitrogen-fixing symbionts in specialized root structures, ensures the selection of efficient nitrogen-fixing strains while limiting less cooperative symbionts.^37^ The specificity of this symbiosis is further shaped by the fact that rhizobia have evolved mechanisms to evade activation of the plant immune system. In particular, they lack key microbe-associated molecular patterns, which are short conserved peptides such as the flg22 epitope in flagellin, and exploit the legume’s inability to detect other conserved bacterial signals like the elf18 peptide from the elongation factor Tu and the csp15 peptide from cold-shock protein.^30^ This immune evasion contributes to host selectivity, favoring beneficial rhizobia and limiting investment in noncooperative strains.^38^

#### Hawaiian Bobtail Squid and his Flashlight *Aliivibrio fischeri*

Another well-characterized model of biochemically mediated mutualism is the binary symbiosis between *Aliivibrio fischeri* and the Hawaiian bobtail squid (*Euprymna scolopes*). After hatching, the squid horizontally acquires the symbiont from seawater, which then colonizes the epithelial crypts of the light organ. In this mutualism, *A. fischeri* benefits from a nutrient-rich habitat, while its bioluminescence, regulated via acyl-homoserine lactones-quorum sensing, provides counterillumination, masking the squid’s shadow at night and enhancing its camouflage from predators and prey.^39^ As in the case of specialized root nodules, the host employs a similar strategy to regulate symbiont interactions by compartmentalizing beneficial microbes within the dedicated light organ, thereby minimizing competition with faster-growing but less beneficial microorganisms.^24,40^ In addition, the host-symbiont specificity is orchestrated through an exchange of chemical signals, including bacterial peptidoglycan, tracheal cytotoxin,^41^ and exopolysaccharides,^42,43^ along with host-derived nitric oxide,^44^ and chitin.^42,45^ Importantly, daily expulsion of 95% of the bacterial population imposes strong selection for traits like synchronized luminescence, favoring phenotypes that optimize quorum sensing-regulated luciferase expression.^46^

One fundamental small molecule detected as involved in the process of daily acquisition of the symbiont is the cyclo­(d-histidyl-l-proline) (**7**), which has been molecularly demonstrated to be fundamental in the establishment as well as maintenance of the symbiosis.^47^ As with the legume-rhizobium symbiosis, initial interactions between *E. scolopes* and *A. fischeri* require a complex host-symbiont dialogue, which is partially already characterized. Clearly, many questions remain unanswered, including some that must await further tool development.

#### Fungus-Growing Ants Ecosystems

Fungus-growing ants, such as those in the genus *Apterostigma*, and their symbiotic *Pseudonocardia* bacteria have become a model system in chemical ecology and a rich source of natural small molecules. In this complex and multilateral mutualism, the ants cultivate specialized fungal crops by supplying plant material, which the fungi degrade to provide nutrients to the ants. This system is challenged by parasitic fungi, particularly *Escovopsis* species, which threaten the cultivated fungus.^48^ To defend against these pathogens, the ants harbor antibiotic-producing *Actinomycete*, mainly *Pseudonocardia*, with which they share a long coevolutionary history.^28^ These bacteria reside in specific niches, on the ant cuticle and are vertically transmitted between generations.^49^ They produce a diverse chemical arsenal of antifungal and antibacterial compounds, including metabolites synthesized via NRPS and PKS pathways, such as dentigerumycin A (**8**),^50^ selvamicin (**9**),^51^ rebeccamycin (**10**),^52^ attinimicin (**11**),^53^ antimycin A1 (**12**),^54^ candicidin (**13**),^55^ valinomycin (**14**),^56^ burkholdine1213 (**15**)^57^ and pyrrolnitrin (**16**),^57^ each contributing to the maintenance and stability of the intricate, multilateral symbiosis. Although other chemically diverse molecules have been reported, no biological or functional role can be always easily established in the fungus-ants ecological context and remain to be investigated. This is the case of pseudonocardones (**17**),^58^ where glycosylation might explain the lack of biological activity in the reported bioassays, which could be a mechanism of self-resistance.^59,60^ Additionally, the role of small molecules as mediators of defense response might be a mechanism involved in structuring symbiotic interactions.^61^ The specific signaling molecules exchanged among microbial communities and their host to establish symbiosis remain unknown, likely due to the vertical mode of transmission. Additionally, chemical reactions (*e*.*g*., biotransformation, metabolism, degradation) can occur within these ecosystems by considering their chemical complexity as molecules originate from plant tissues brought by the ants, but also from opportunistic pathogens.^61^ Spatial distribution of molecules can accelerate the discovery of molecules involved in symbiosis by revealing chemical modifications occurring *in situ*.^62^

#### Human Gut and the Microbiome

The concept of symbiosis becomes substantially more complex when applied to mammals, whose microbiota encompasses hundreds of interacting taxa and dynamically interfaces with host physiology across multiple systems. In contrast to well-characterized examples of obligate endosymbionts in invertebrates, where symbiont genome reduction and vertical transmission offer clear signatures of coevolution, the mammalian microbiota is largely horizontally acquired, compositionally flexible, and functionally redundant, posing significant challenges to defining it as a classical example of symbiosis. Consequently, comprehensive examples of host–symbiont coevolution in mammals have remained relatively scarce. However, recent advances in microbiome research are reshaping our understanding of species evolution, highlighting the gut microbiota as a central player in host adaptation and health. Beyond traditional views of mutualism, emerging evidence suggests that host–microbiota interactions represent a multilayered coevolutionary process involving a stable, host-adapted microbial core and a flexible, environmentally modulated pool.^3^ Among the dominant members of this core microbiome, *Bacteroides* spp., comprises up to 50% of cells in the Western adult gut microbiota, playing a pivotal role in shaping immune development and maintaining intestinal homeostasis. Notably, *Bacteroides fragilis* produces polysaccharide A, a zwitterionic capsular polysaccharide that directly modulates host immunity by promoting CD4^+^ T cell development and restoring TH1/TH2 balance in germ-free mice, functioning as a prototypical “symbiosis factor” essential for proper immune organogenesis.^63^ In parallel, *Bacteroides thetaiotaomicron* has been shown to synthesize sphingolipids, including ceramide phosphoethanolamine (**18**), that are critical for intestinal homeostasis; mice colonized with a sphingolipid-deficient mutant exhibit inflammatory phenotypes, including crypt hyperplasia and macrophage infiltration, underscoring the immunomodulatory potential of these bacterial lipids.^64^ Furthermore, *B. thetaiotaomicron* produces a structurally unique lipooligosaccharide with a penta-acylated lipid A core (**19**), distinct from the proinflammatory lipopolysaccharide of pathogens. This modified lipooligosaccharide avoids triggering TLR4-mediated responses and may contribute to immune tolerance toward *Bacteroidetes* as well as other symbionts.^65^ Together, these findings highlight *Bacteroides* not only as structural and metabolic contributors to the gut ecosystem, but also as a possible human-symbiont able to synthesize natural products that strengthen the host immunity and contribute to the host health status. Thus, it is time to reconceptualize the mammalian host not as a solitary entity but as a holobiont, with the microbiome acting as a symbiotic organ system, which have undergone reciprocal selection shaping immunological, metabolic, and developmental pathways. We hypothesize that several other members of the gut core microbiome may play key functional roles comparable to those of *Bacteroides*; however, current data are insufficient to substantiate this hypothesis.

After this overview of symbiotic examples, a fundamental question remains: how many other natural products are we currently overlooking due to limitations in experimental resolution or ecological context? We believe that small-molecule chemistry will be imperative in studying and testing complex host-bacterial relationships, also to discover novel bioactive molecules that are the result of shared and complementary biosynthetic pathways.

### The Unknown Metabolome, Potential for Discovery of Molecules Involved in Symbiosis

Bacteria are a major source of bioactive natural products. Although the vast majority of known bacterial metabolites have been identified and studied in free-living organisms, there is growing evidence that bacteria engaging in symbiotic relationships exhibit remarkable chemical productivity and harbors a rich reservoir of novel biosynthetic enzymes and secondary metabolites.^86^ However, the discovery of molecules involved in symbiotic interactions is hindered by several key limitations that span from the biological complexity of the symbiosis relationship, including technical challenges to recapitulate such relationship *in vitro* to the lack of up-to-date technologies.

Many symbiosis-associated molecules are tightly regulated and produced only under specific conditions, making their detection difficult in standard *in vitro* systems that fail to replicate the natural context. Several natural products identified to date as important in symbiosis have been biologically characterized outside of their native context, typically under controlled laboratory conditions. As a result, directly linking these compounds to specific biological functions within the symbiotic interaction remains challenging, based solely on chemical structure.^87^

It is essential to move beyond traditional *in vitro* experimental systems and explore more ecologically realistic settings. Spatial distribution, or spatial proximity between host and symbiont and time-resolved sampling in natural or seminatural environments offer powerful strategies to capture the dynamic nature of molecular interactions between host and microbe(s). While small molecules were often prioritized for isolation and structural elucidation due to their high abundance and relative ease of detection, this scenario is not necessarily valid today. The expression of several biosynthetic gene clusters (BGCs) from *Streptomyces* spp. are tightly regulated via environmental cues and small-molecule autoregulators,^88^ which result in overlooking a wide array of context-dependent molecules. Moreover, these natural products are often present in low abundance or expressed transiently, requiring spatially and temporally resolved sampling strategies for effective identification. Without accounting for these variables, it becomes difficult to link molecular presence to functional relevance within the symbiotic relationship.

By looking at the marine environments, studies on the coral holobiont have shown that abiotic factors such as day light can strongly influence microbial metabolism. Moree and colleagues demonstrated that a *Pseudoalteromonas* strain, isolated from healthy octocoral tissue, produces antifungal polyketides, specifically alteramides, at significantly higher levels in the dark. Using MALDI-IMS, they found that light triggers a photoinduced intramolecular cyclization of the alteramides, rendering them inactive. These findings suggest a light-dependent regulatory mechanism, where enhanced production of active antifungal compounds in the dark may help protect corals - which are nocturnal feeders—from fungal pathogens during nighttime when they are more susceptible to infection.^89^ This is an example of the importance of investigating the host-microbe symbiosis directly from their ecological context.

In addition, it is crucial to recognize that microbes live within highly complex communities, where interactions among multiple members can significantly influence the symbiotic outcome of a specific microbe–host relationship, which are typically oversimplified in binary host–microbe models. The metabolic output of a microbe changes drastically between monoculture conditions and natural setups where interactions with the host and other members of a microbial community occur.^90^ Such ecological complexity strongly influences gene expression and metabolite production, explaining why many BGCs remain unexpressed under standard laboratory conditions. Moreover, several cell-to-cell communication mechanisms, such as quorum sensing, occur in these complex systems, which are crucial for regulating gene expression in a density-dependent manner and often serve as the triggers for specialized metabolite biosynthesis.^91^ In the absence of these natural chemical signals, many compounds are never produced, leaving a large portion of microbial chemical diversity unexplored.

This challenge is compounded by the fact that most symbiotic microbes remain uncultivated, restricting experimental access to their biosynthetic potential. Even when BGCs are discovered through genome mining, identifying the environmental or molecular cues that activate them remains a major bottleneck, particularly for silent or cryptic pathways. To overcome these limitations, *in situ*–like cultivation strategies that use host-derived culture media and integrate multiomics data are becoming essential. By replicating the native physiological context of microbial communities, these approaches, together with advances *in vivo* models and tissue culture systems, are facilitating the cultivation of previously unculturable microorganisms. Concurrently, progress in metagenomics and metatranscriptomics continues to reveal the structure and function of microbiomes and symbiotic associations, offering deeper insight into the molecular basis of host–microbe interactions.^92–94^

As such, a holistic and systems-level approach is essential for advancing our understanding of symbiosis. This need is particularly evident in studies of the human and plant microbiomes, where close host associations emerge from the integration of diverse biochemical pathways and multilayered regulatory interactions across microbial consortia.

To effectively explore these dynamics, symbiosis research must increasingly rely on comprehensive omics-based methodologies, including metabolomics, proteomics, transcriptomics, metagenomic library construction and screening, heterologous expression, community sequencing, and single-cell technologies. The integration and interpretation of these complex data sets are being greatly accelerated by recent advances in artificial intelligence and machine learning, offering powerful tools for uncovering novel mechanisms and driving discovery in the field. However, the lack of multiomics and well-curated comprehensive data sets to enable such applications is a current limitation.

From the metabolomics field, a significant frontier in the discovery of symbiosis-related natural products lies within the dark metabolome.^95^ Reverse metabolomics^96^ and data mining approaches^97,98^ leveraging recent tools and publicly available metabolomics data sets, have demonstrated how powerful these resources can be for discovery of natural products. Together, they offer a path to accelerate our understanding of small-molecule–mediated symbioses by allowing the interrogation of entire metabolomes rather than individual compounds. To explore chemical interactions between organisms, metabolomics approaches offer a starting point, employed through either untargeted or targeted approaches. Untargeted metabolomics captures a broad overview of all detectable metabolites present in a sample under specific experimental conditions. However, the exact set of metabolites detected can vary depending on factors such as the extraction protocol, data acquisition method, and analytical instrumentation. Advancements in data-independent acquisition techniques have enhanced the ability to detect a wider range of metabolites and lowered detection limits, particularly in the context of natural product discovery. In contrast, targeted metabolomics focuses on quantifying a specific set of known metabolites, typically to test or validate a hypothesis. Together, these two approaches play complementary roles in generating, refining, and validating hypotheses, ultimately shedding light on underlying biochemical processes.

In recent years, online MS/MS databases have rapidly expanded in terms of library size and associated tools, due to openly available, user-submitted MS data, and advances in computational power and machine learning-based analytical techniques in the analysis of mass spectrometry data. The higher sensitivity of MS enables detection of specialized metabolites, which are usually found in low concentrations. Besides identifying the already detected molecules, such as the thousands of unknown microbial natural products (Figure 2), the next step leads to confirm whether these molecules or their analogues (*e*.*g*., chemical modifications, biotransformation or degradation products only observed in the symbiotic system) are observed in natural environments, therefore the need of environmental samples. Once confirmed, these findings may indicate functional or ecological roles among these molecules and, more broadly, offer valuable clues for developing new hypotheses about their ecological significance. In the next section, we offer our perspective and suggest mass spectrometry-based workflows to reveal molecules involved in symbiosis, so future research will provide symbiosis score 3 for most of the natural products known today and for those to be discovered.

**Figure 2:**
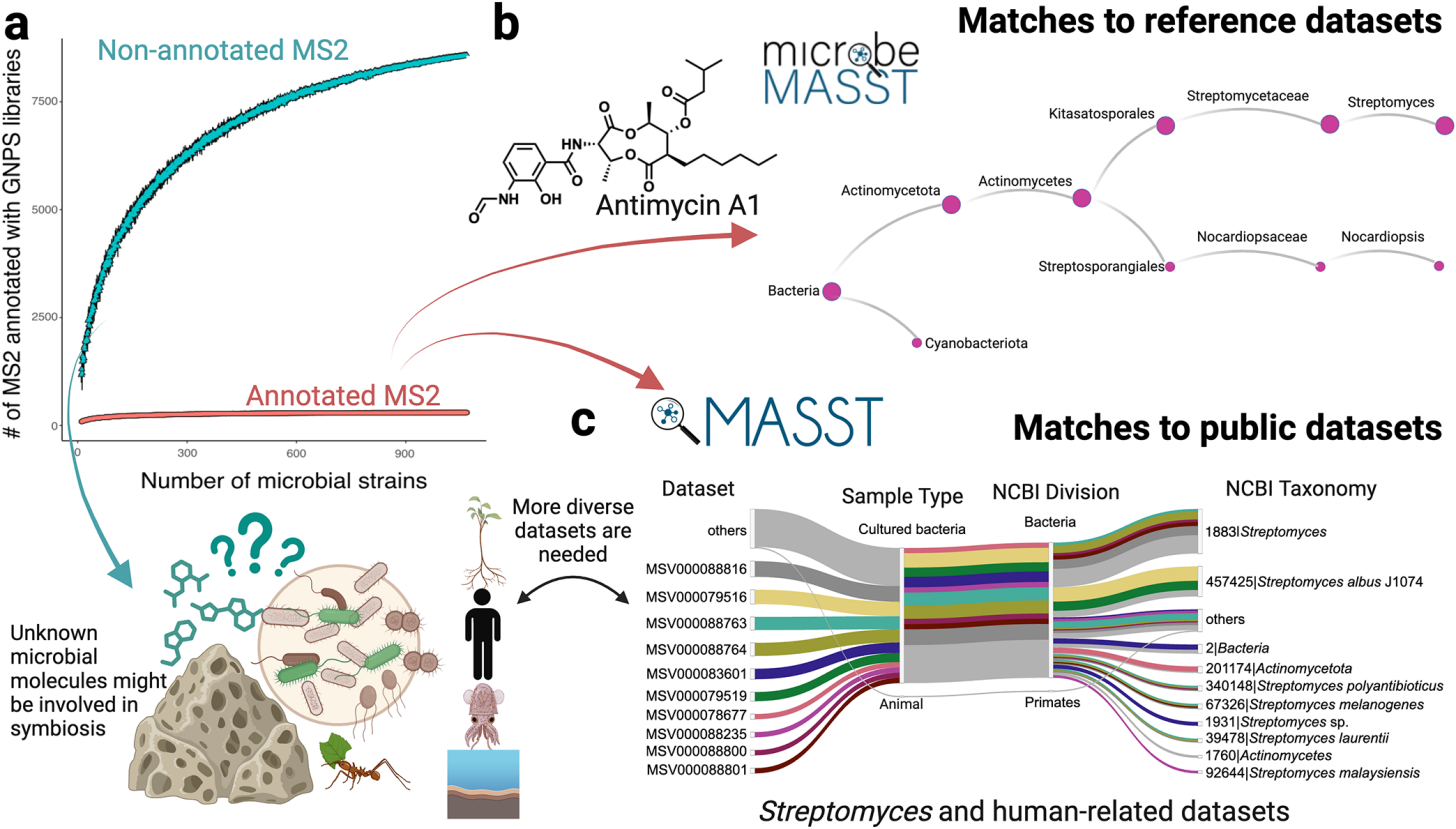
Unknown microbial natural products, a reservoir of molecules involved in symbiosis to be discovered. (a) Rarefaction analysis performed on fragmentation spectra from microbial strains annotated with GNPS2 libraries^66^ (annotated MS2) while the remaining (nonannotated MS2) indicate the potential for discovery that this and other public data sets contain.^67^ Briefly, in the rarefaction analysis, each MS/MS spectrum in the data of a sample is counted, then for the next sample each additional MS/MS that is not found in the data from the first sample is counted, and then for the third sample, data will be added but only if the MS/MS is not found in the first two samples. Rarefaction is continued in this fashion until all the MS/MS are found in the entire data; (b) fragmentation spectra can be searched across reference microbial data sets using microbeMASST,^68^ providing evidence of its biological origin. Antimycin A1­(**12**) is a depsipeptide biosynthesized by a NRPS-PKS system in *Actinomycetes*.^54^ These versatile microorganisms are present in diverse environments, such as plant and soil ecosystems, suggesting microbial molecules such as antimycins can be potentially involved in symbiosis; (c) by using Mass Spectrometry Single Search Tool (MASST),^69^ molecules can be searched across repositories,^66,70–72^ providing results indicating whether the target molecule is present in public data sets of microorganisms,^68^ or even animal tissues.^73^ Molecules are found in public data sets and if so, it is possible to retrieve all available information associated with the data set, such as taxonomy (*e*.*g*., genus). Results from MASST search were visualized as a Sankey plot where each line color corresponds to a different public data set. Matches of antimycin were found in microbial data sets mostly from *Actinomycetes*,^74–83^ while matches to human fecal samples are from the American Gut Project.^84,85^ Finally, the more diverse data sets are available, as highlighted under the rarefaction analysis (a), the likelihood of finding additional sources of the target molecule increases. Figure partially created with BioRender.

### How to Discover Molecules Involved in Symbiosis

By considering the rapid access to data from untargeted approaches, such as tandem mass spectrometry, several resources can be leveraged to reveal small molecules involved in symbiotic relationships. With the availability of environmental and biological samples, one can start hypothesis-generating approaches: are the detected molecules produced by the microbial symbiont, by the host or by both, symbiont-host? If the last is true, under which conditions these molecules are produced? And which is their biological function? These questions fit in the classical approach from the natural products discovery field, prioritizing molecules based on chemical novelty and biological origins. Now, from the symbiosis investigation, the challenge goes about confirming the role of these molecules in establishing or maintaining the symbiont-host relationship. To answer these questions, several approaches can be taken as proposed in the following steps. By leveraging mass spectrometry-based approaches (Figure 3a), it would be possible to confirm the role of natural products in symbiosis, as exemplified by cyclo­(d-histidyl-l-proline) (**7**) (Figure 3b):

1. ***Data collection from biological samples and environments***: Once molecules are detected from biological samples and environments (metabolomics approach), their unique fragmentation patterns (MS/MS) can be used to search across repositories.^66,70–72^ Hence, the importance of making the data public, so the community can leverage the data sets while contributing to its investigation. Once the data is deposited in public repositories, it will also contribute with the repository scale search for other molecules.^66,99^ ***Current limitations***: A possible limitation in the data collection from underexplored ecosystems or organisms might come from the actual sample collection. Noninvasive options, for instance when collecting marine organisms such as coral tissues, might not be possible yet. On the other hand, collecting data from environmental samples is possible and sampling devices and protocols are continuously evolving.^100,101^

2. ***Curating associated information and preparing metadata***: The quality and accuracy of metadata are what allow scientists and the broader community to perform analyses across individual studies and large-scale repositories. Identifying the presence of a molecule in a biological sample and linking the associated information such as sample type, species, or environment, allows researchers to assess how specific a molecule is and to hypothesize their potential involvement in symbiosis. This can be performed by having correct metadata associated with the metabolomics data sets, and leveraging the Pan-ReDU infrastructure that enable the investigator to generate and test these hypothesis.^99^ The Pan-ReDU infrastructure includes a metadata template with controlled vocabularies and ontologies to facilitate standardization across data sets from diverse sources (e.g., repositories, instruments, biological samples). This effort facilitates data reuse, so scientists can connect information and hypothesize if a molecule is specific to an organism or environment. This kind of analysis can be performed directly in the Pan-ReDU infrastructure.^99^ ***Current limitations***: Although awareness to contribute is growing across the scientific community, there is still no complete consensus regarding standard metadata for metabolomics. However, templates and standard vocabulary has been provided in recent publications to address this challenge.^99,102^

3. ***Chemical relationship and library identification***: This is where molecular networking and library search,^66,103^ together with recently developed tools and infrastructures such as MASST (Mass Spectrometry Single Search Tool) domain specific tools (*e*.*g*., microbeMASST)^68,69^ and Pan-ReDU^99^ can be leveraged. Once molecules are found in public data sets, the next step is to confirm the molecule is known and available. This means, the molecule(s) of interest can be found commercially available, isolated from biological sources and/or synthesized, then confirmed by orthogonal approaches (e.g., liquid chromatography for retention time comparison). ***Current limitations***: A bottleneck in metabolomics relies on the lack of reference spectra. Even if library annotations are obtained, stereochemistry cannot be confirmed only by mass spectrometry. Therefore, spectral libraries accelerate the process of identifying molecules, but additional steps are needed (e.g., synthesis or isolation for full chemical characterization).
4. ***MASST, Single search approaches***: Molecule search is possible across reference data set or at repository scale. The MASST tool^69^ enables the investigator to search fragmentation spectra of molecules of interest across data sets. This is a broad step, as the results will provide a diversity of data sets where the molecule of interest has been detected. ***Current limitations***: One caveat of this approach relies on the fact that not all deposited data contains associated metadata, as suggested in step 2. However, data sets have contact information and minimal information (*e*.*g*., species or description of experiments), so the researcher can attempt to reach out to depositors regarding additional information associated with the data sets and proceed with further analysis.
5. ***Domain specific MASST***: If the goal is to know whether molecules are of microbial origin, then microbeMASST is an option. An ongoing community effort to create and curate microbial molecules is already available through the Collaborative Microbial Metabolite Center (https://cmmc.gnps2.org/). With the CMMC knowledgebase, one can enrich a molecular network to identify which molecules have been already reported as microbial. With these two approaches, MASST and CMMC, molecules of microbial origin can be captured. ***Current limitations***: An evident limitation of this approach are the amount of reference data sets and reference molecules deposited in such infrastructures. Although growing, researchers require additional stimulus to contribute with these efforts as it can be seen as time-consuming steps with no benefit in the short term. However, performing analysis in minutes that 5–10 years ago were not possible, should compensate for the investment.
6. ***In vivo and in vitro validation***: once molecules have been prioritized using any of the previous steps, validation steps aiming to recapitulate, either partially or completely, the involvement of molecules in a symbiotic relationship can be done. This is not a trivial task, and likely one of the most challenging aspects of this kind of research. Even in apparently “simple” symbiosis, such as the one between *A. fischeri* and the Hawaiian bobtail squid (*E. scolopes*), where there is one symbiont and one host, revealing which molecules are responsible for establishing this relationship can take years of research (Figure 3b).^40,47^ However, this is a very good example for the approach that can be applied when studying more complex systems, as setting up a proper bioassay, either *in vitro* or *in vivo*, from animal models to cell cultures, can provide another layer of uncertainties that need to be confirmed with *in situ* measurements of the molecules of interest. ***Current limitations***: As mentioned, these steps might take years due to several factors, such as sensitivity of the bioassay, spatial resolution, timing or *in vivo* recapitulation of the expected effect. Validating symbiosis-associated molecules remains extremely challenging because their activity is generally context-dependent and emerges only within specific microbial or host–microbe interactions that are difficult to reproduce experimentally (i.e., host developmental stage, immune status, oxygen availability, or physical microstructure). Many symbionts are not genetically manipulable, host conditions can be difficult to reproduce, and the chemistry itself may change once inside the host. These factors make it hard to recapitulate *in vitro* the observations made *in situ* and slow down the confirmation of a molecule’s true role in symbiosis. Another potential limitation, commonly observed in the natural products field, relates to chemical stability of the chemical entity to test. Isolation, purification and chemical characterization steps will provide insights regarding stability of the molecule of interest, however stability *in vivo* might present challenges by itself.
7. ***Reverse metabolomics approaches***: These approaches,^96,104^ which refers to the process of searching fragmentation spectra of molecules (*e*.*g*., synthesized or commercially available) and their presence in biological systems, can be used either as confirmatory step or as a reverse approach, to test whether molecules are involved in symbiosis. This approach can be leveraged to confirm if molecules are of symbiont, host or symbiont-host origin following the previous steps, starting with the already known molecules instead of the complex biological sample. ***Current limitations***: A limitation of this approach relies on the amount of available data from understudied organisms and ecosystems when compared to human and animal models. Therefore, further efforts should be made to the contribution of environmental and understudied organisms and ecosystems, so reverse metabolomics approaches can be leveraged.

**Figure 3:**
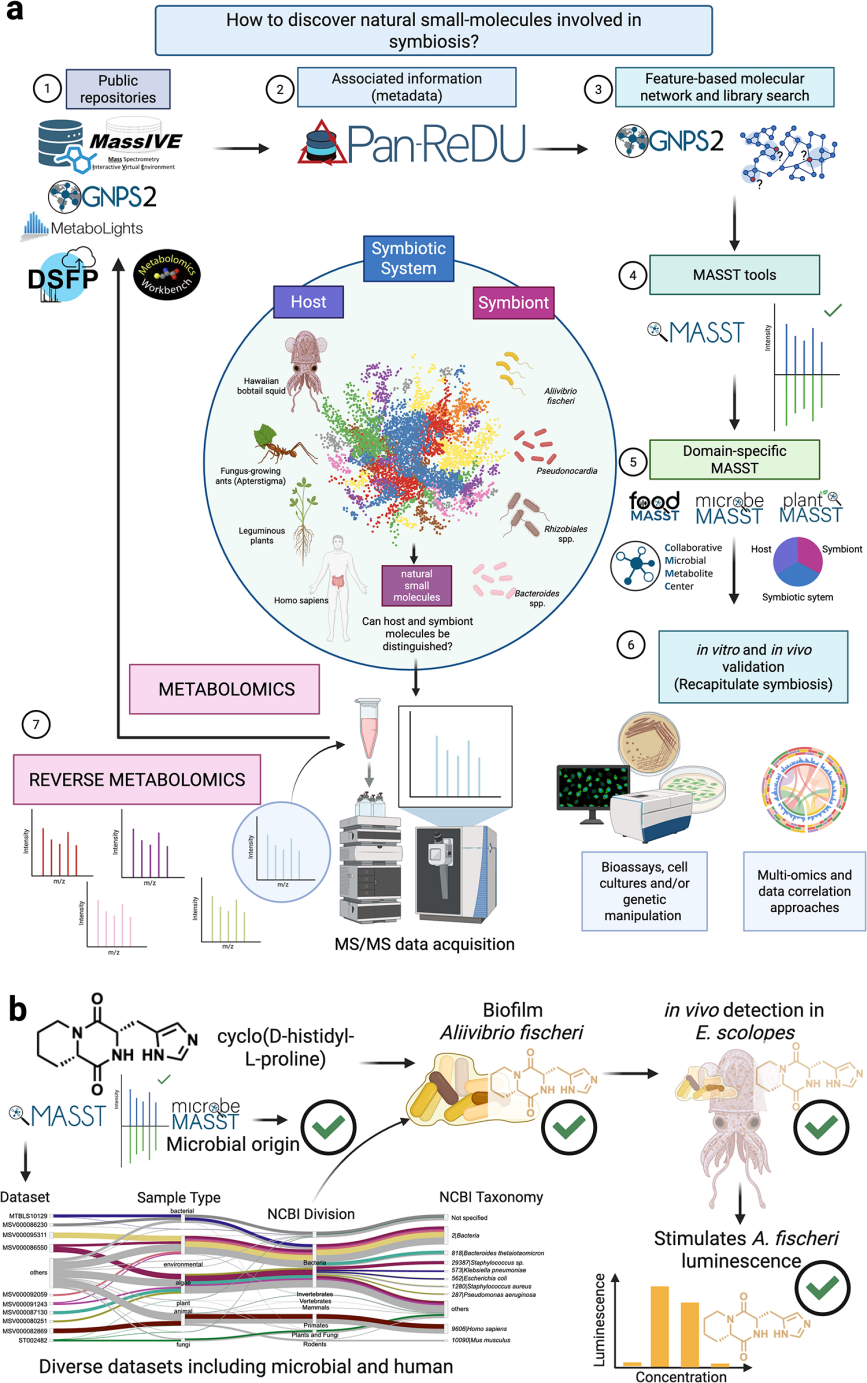
Future perspectives applying MS-metabolomics to discover molecules involved in symbiosis. (a) This proposed workflow consists in the following steps: 1. After data is acquired, it is deposited in public repositories; 2. Associated information is provided following standard vocabulary (ReDU); 3. Molecular Networking and library search approaches provide identification and chemical relationships of detected molecules; 4. MASST tools enable to search molecules based on their fragmentation spectra across entire repositories; 5. Searches can also be performed on domain (*e*.*g*., microbeMASST to find out if a molecule is of microbial origin). The previous steps will provide an overview about the number of spectra present in public repositories, an indication of these molecules to have a potential biological role in the systems they have been observed; 6. After prioritizing molecules for further biological assays (*e*.*g*., quorum sensing, signaling, antimicrobial, biofilm inhibition or formation, etc.), their potential involvement in the symbiotic systems of study can be demonstrated; 7. Reverse metabolomics approaches start by a molecule, instead of a biological sample as traditional metabolomics approaches, and search that molecule in public repositories, and continue leveraging the previous steps to demonstrate the involvement of molecules in symbiosis; (b) by using cyclo­(d-histidyl-l-proline) (**7** in Figure 1) as one of the few examples found in literature where the involvement of microbial molecules has been demonstrated in symbiosis, including *in vivo* detection of the microbial molecule,^47^ we highlight the steps suggested in panel **a** as a useful workflow to discover other molecules involved in symbiosis from more complex ecosystems. By using MS/MS data (Step 1–3 in a), the molecule of interest can be searched across public repositories using MASST (Step 4 in a). This diketopiperazine has been reported from bacteria, fungi, squids and human samples. By searching this molecule at the repository scale, it was found in diverse data sets from bacteria, fungi and human samples (colors in Sankey plot indicate different data sets).^106–115^ By using microbeMASST (Step 5 in a), one can suggest potential microbial origin that can be confirmed by microbial monocultures of a selected strain (*A. fisheri* in this case). Once microbial origin is confirmed, *in vivo* assays (Step 6 in a) can be used to recapitulate and confirm the presence of the molecule in the symbiotic system. Finally, it is possible to demonstrate a role of the molecule by selecting a specific assay, in this example an increase of luminescence in *A. fisheri* in a concentration-dependent manner was demonstrated.^47^ Figure created using BioRender.

Finally, several resources are already available for the community to use and apply to their own research. To facilitate data processing of results from the workflows mentioned above, including access to repository-scale analysis, to the broader community, several apps are already available and suitable for small molecule-related hypotheses to test using metabolomics approaches.^105^

## Conclusion

We have provided an overview of natural products research merged into the study of symbiosis, focusing on bacterial associations with plants, insects, marine organisms, and humans. An existing gap between the already known (and unknown) chemical space of natural products and their role in symbiosis is evident. Even relatively “simple” symbiotic systems where one symbiont and one host establish a sustainable and vital relationship, such as the one observed between *A. fischeri* and the Hawaiian bobtail squid (*Euprymna scolopes*), have resulted in the confirmation of one molecule involved in the establishment of such symbiosis.^47^ With the ongoing advances in the field of metabolomics, and how the analysis of small molecules and metabolomes will accelerate the understanding of symbiotic systems, we suggest steps that leverage current resources, with an emphasis on the availability of public metabolomics data sets. By providing associated information about biological origin, sample type, collection sites and any additional information that facilitate the recapitulation of proper conditions where molecules are produced, the role of such molecules will become evident. Data analysis at the repository scale is now available,^99^ however strong conclusions and even confirmatory analysis still rely on the availability of reference and well-curated data sets. With that in mind, and the aim of revealing whether molecules are specific to one symbiotic system or general across systems, or even whether a molecule plays a role in one system while playing a completely different or opposite role in another system remain questions that will be answered in the next decade of research, and we hope mass spectrometry-approaches will enable us to accelerate the pursuit of such answers.

## Supplementary Material



## Notes

List of 94 natural products involved in symbiosis and includes molecule’s name, symbiosis score, host, symbiont symbiosis involvement and references (Table S1) (PDF)
