# Supplementary material for: From Molecules to Metabolomes, Understanding Symbiosis through Small Molecules
Source: J Nat Prod. 2026 Mar 18;89(4):1103–16. doi: 10.1021/acs.jnatprod.5c01360 (PMC13122640; doi:10.1021/acs.jnatprod.5c01360)
Supplement: Supplementary file 1 [file np5c01360_si_001.pdf]

Supporting Information

From molecules to metabolomes, understanding symbiosis through small molecules

Cristina Bez[1,2], Yasin El Abiead[3], Andrés M. Caraballo-Rodríguez*[1]

[1]Skaggs School of Pharmacy and Pharmaceutical Sciences, University of California San Diego, 9500 Gilman Drive, San Diego, California, 92093-0751, United States, [2]Bacteriology Group, International Centre for Genetic Engineering and Biotechnology (ICGEB), Padriciano 99, Trieste, 34149, Italy, [3]Institute of Analytical Chemistry, Department of Chemistry, University of Natural Resources and Life Sciences, Vienna, Vienna 1190, Austria

Author Information

Corresponding Author

Andrés M. Caraballo-Rodríguez - Skaggs School of Pharmacy and Pharmaceutical Sciences, University of California San Diego, 9500 Gilman Drive, San Diego, California, 92093-0751, United States; https://orcid.org/0000-0001-5499-2728; Email: acaraballorodriguez@health.ucsd.edu

Authors

Cristina Bez - Skaggs School of Pharmacy and Pharmaceutical Sciences, University of California San Diego, 9500 Gilman Drive, San Diego, California, 92093-0751, United States; Bacteriology Group, International Centre for Genetic Engineering and Biotechnology (ICGEB), Padriciano 99, Trieste, 34149, Italy; https://orcid.org/0000-0002-9418-7923; Email: cristina.bez@icgeb.org

Yasin El Abiead - Institute of Analytical Chemistry, Department of Chemistry, University of Natural Resources and Life Sciences, Vienna, Vienna 1190, Austria; https://orcid.org/0000-0003-4392-7706; Email: elabiead@gmail.com

Table of Contents

Table S1. Small molecules involved in symbiosis **S2**

References **S5**

Table S1. Natural Products involved in symbiosis

Molecule	Symbiosis score*	Host	Symbiont	Symbiosis involvement	Reference
2,3-Indolinedione (Isatin)	1	shrimp *Palaemon macrodactylus*	*Alteromonas sp.*	Maintenance	[1]
Psymberin	1	Psammocinia aff. bulbosa sponge	*cyanobacteria (Tychonema, Theonella, Oscillatoria etc)*	Maintenance	[2]
Saxitoxin	1	dinoflagellates	*cyanobacteria (Anabaena, Aphanizomenon)*	Maintenance	[3–5]
Tetrodotoxin	1	pufferfish, newts, octopus	*Vibrio, Pseudomonas, Alteromonas, etc.*	Maintenance	[6]
Dimethylsulfoniopropionate	1	coral	*Endozoicomonas*	Establishment	[7]
Guaiacol (2-Methoxyphenol)	1	locusts	*Pantoea agglomerans*	Maintenance	[8]
Formic Acid	1	honeybees	*LAB (Lactobacillus & Bifidobacterium)*	Maintenance	[9]
Lactic Acid	1	honeybees	*LAB (Lactobacillus & Bifidobacterium)*	Maintenance	[9]
Hydrogen Peroxide	1	honeybees	*LAB (Lactobacillus & Bifidobacterium)*	Maintenance	[9]
2-Heptanone	1	honeybees	*LAB (Lactobacillus & Bifidobacterium)*	Maintenance	[9]
Benzene	1	honeybees	*LAB (Lactobacillus & Bifidobacterium)*	Maintenance	[9]
Mycangimycin	1	Southern Pine beetles (*Dendroctonus frontalis*)	*Actinobacteria (unspecified)*	Maintenance	[10]
Formononetin	1	soybean (Glycine max)	*arbuscular mycorrhizal fungi*	Establishment	[11]
Rhizoxin	1	*Rhizopus microsporus* (fungus)	*Burkholderia rhizoxinica*	Maintenance	[12]
Bryostatins	2	*Bugula neritina*	*Endobugula sertula*	Maintenance	[13–16]
Dysideathiazole And Dysidenin	2	marine sponge (*Lamellodysidea herbacea*)	*cyanobacterium, Oscillatoria spongeliae*	Maintenance	[17,18]
Onnamide A	2	marine sponge *Theonell*a sp.	*Candidatus Entotheonella*	Maintenance	[19]
Polytheonamides	2	marine sponge *Theonella* sp.	*Candidatus Entotheonella*	Maintenance	[19,20]
Theopederin	2	marine sponge *Theonella* sp.	*Candidatus Entotheonella*	Maintenance	[19]
Indigoidine	2	bobtail squid	*Leisingera sp. JC1*	Maintenance	[21,22]
Daunomycin	2	deep-sea coral invertebrates/intertidal macroalgae	*Streptomyces cyaneofuscatus*	Maintenance	[23]
Cosmomycin B	2	deep-sea coral invertebrates/intertidal macroalgae	*Streptomyces cyaneofuscatus*	Maintenance	[23]
Galtamycin B	2	deep-sea coral invertebrates/intertidal macroalgae	*Streptomyces cyaneofuscatus*	Maintenance	[23]
Maltophilin	2	deep-sea coral invertebrates/intertidal macroalgae	*Streptomyces cyaneofuscatus*	Maintenance	[23]
Iobophorine B	2	deep-sea coral invertebrates/intertidal macroalgae	*Streptomyces carnosus*	Maintenance	[23,24]
Germicidin A-B	2	deep-sea coral invertebrates/intertidal macroalgae	*Streptomyces carnosus*	Maintenance	[23]
Nitric Oxide	2	*Trichodesmium erythraeum* (algae)	*Silicibacter sp.*	Both	[25]
Nitric Oxide	2	*Euprymna scolopes*	*Vibrio fischeri*	Establishment	[26]
Thallusin	2	Ulva and Enteromorpha marine algal genera (alga *Monostroma oxyspermum*)	*epiphytic marine bacterium*	Maintenance	[27]

Molecule	Symbiosis score*	Host	Symbiont	Symbiosis involvement	Reference
Tambjamine	2	*Ciona intestinalis*	*Pseudoalteromonas tunicata*	Maintenance	[28]
Amicoumacins A-C	2	*Steinernema nematodes*	*Xenorhabdus bovienii*	Maintenance	[29]
Xenocoumacins 1-2	2	*Steinernema carpocapsae*	*Xenorhabdus nematophila*	Maintenance	[30]
Darobactin	2	nematodes *Heterorhabditis* spp.	*Photorhabdus khanii HGB1456*	Maintenance	[31]
Rhabduscin	2	Nematodes and insect larvae	*Photorhabdus luminescens and Xenorhabdus spp.*	Maintenance	[32]
Pederin	2	beetle *Paederus sabaeus/Theonella swinhoei* sponge	*Pseudomonas sp.*	Maintenance	[33]
Dentigerumycin A	2	ant *Apterostigma dentigerum*	*Pseudonocardia*	Maintenance	[34]
Gerumycin A-C	2	ant *Trachymyrmex cornetzi*	*Pseudonocardia*	Maintenance	[35]
Selvamicin	2	ant *Apterostigma dentigerum*	*Pseudonocardia*	Maintenance	[36]
Rebeccamycin	2	ant *Apterostigma dentigerum*	*Pseudonocardia*	Maintenance	[37]
Antimycin A1	2	ant *Acromyrmex octospinosus*	*Streptomyces sp.*	Maintenance	[38]
Coprisidin A	2	beetle *Copris tripartitus*	*Streptomyces sp*	Maintenance	[39]
Streptochlorin	2	beewolf digger wasps (*Philanthus* spp. Hymenoptera, Crabronida	*Candidatus Streptomyces philanthi*	Maintenance	[40,41]
Piericidins A1 And B1	2	beewolf digger wasps (*Philanthus* spp. Hymenoptera, Crabronida	*Candidatus Streptomyces philanthi*	Maintenance	[41]
Actinomycins	2	beewolf digger wasps (*Philanthus* spp. Hymenoptera, Crabronida	*Candidatus Streptomyces philanthi*	Maintenance	[42]
Valinomycin	2	beewolf digger wasps (*Philanthus* spp. Hymenoptera, Crabronida	*Candidatus Streptomyces philanthi*	Maintenance	[43]
Colibactin	2	honey bee, *Apis mellifera* gut microbiome	*Frischella perrara*	Maintenance	[44]
Genistein	2	soybean (*Glycine max* L.)	*Bradyrhizobium diazoefficiens USDA110*	Establishment	[45]
Rhizobitoxine	2	*Macroptilium atropurpureum*	*Bradyrhizobium elkanii*	Maintenance	[46]
Benzoxazine	2	maize	*root/rhizosphere microbiome*	Establishment	[47]
Daidzein	2	soybean (*Glycine max* L.)	*Bradyrhizobium japonicum*	Establishment	[48]
Stachydrine	2	*M. sativa* L.	*Rhizobia*	Establishment	[49]
Trigonelline	2	*M. sativa* L.	*Rhizobia*	Establishment	[49]
Jasmonic Acid	2	*Medicago truncatula*	*Sinorhizobium meliloti 1021 (Sm1021)*	Both	[50]
Chloropreussomerins A-B	2	*Acanthus ilicifolius* mangrove	*Lasiodiplodia theobromae (fungal endophyte)*	Maintenance	[51]
Daldionins	2	orchid root	*root-associated symbiont Daldinia eschscholtzii (a fungus)*	Maintenance	[52]
Nodulones B-C	2	orchid root	*root-associated symbiont Daldinia eschscholtzii (a fungus)*	Maintenance	[52]
Nodularin	2	cycad plants	*cyanobacteria (Nodularia spumigena,Nodularia sphaerocarpa PCC7804)*	Maintenance	[53]

Molecule	Symbiosis score*	Host	Symbiont	Symbiosis involvement	Reference
β-N-methylamino-L-alanine	2	flowering plant *Gunnera monoica*/cycad plant, lichen - /Liverworth/Hornworth/	*cyanobacteria (Nostoc)*	Maintenance	[54]
Oxylipins	2	anemone *Exaiptasia diaphana*	*Breviolum minutum (dinoflagellate)*	Maintenance	[55]
Bacteriocin	2	hoopoe birds (*Upupa epops*) - uropygial gland	*Enterococcus faecalis*	Maintenance	[56,57]
Lactocillin	2	human vaginal microbiota	*Lactobacillus gasseri*	Maintenance	[58]
Marinomycins	2	human oral microbiome	*Propionibacterium propionicum and Actinomyces timonensis*	Maintenance	[58]
Humimycin A and B	2	human nasal, oral, eye microbiome	*Rhodococcus equi and R. erythropolis*	Maintenance	[59]
Colibactin (Precolibactin)	2	human gut microbiome	*E. coli* (including Nissle 1917), *Klebsiella,* etc.	Unclear	[60]
Pyridoxatin	2	*Lethariella zahlbruckner* - lichen	*Tolypocladium cylindrosporum*	Maintenance	[61]
Microcystin	2	lichen	*cyanobacteria (Nodularia)*	Maintenance	[62]
Nosperin	2	lichen - Peltigera	*cyanobacteria*	Maintenance	[63]
Uncialamycin	2	lichen *Cladonia uncialis*	*Streptomyces uncialis*	Maintenance	[64]
Coumabiocins A–F	2	lichen *Cladonia gracilis*	*Streptomyces sp. L-4-4*	Maintenance	[65]
Valinomycin	2	*Acromyrmex echinatior* and *A. niger*	*Streptomyces spp.*	Maintenance	[66]
Pseudonocardones A-C	2	*Apterostigma dentigerum*	*Pseudonocardia spp.*	Maintenance	[67]
Diketopiperazine, Cyclo(D-Histidyl-L-Proline)	3	Hawaiian bobtail squid *Euprymna scolopes*	*Vibrio fischeri*	Establishment	[68]
Roseobacticides A-K	3	*Emiliania huxleyi*	*Phaeobactergallaeciensis BS107*	Establishment	[69,70]
Cyanobactins (Patellamides, Trunkamide, Patellins)	3	*Lissoclinum patella* (ascidian)	*Prochloron spp.*	Maintenance	[71,72]
Bromoalterochromides A-B	3	Hawaiian bobtail squid *Euprymna scolopes*	*Pseudoalteromonas sp. JC28*	Maintenance	[22]
Kahalalides	3	Hawaiian marine alga *Bryopsis* sp.	*Candidatus Endobryopsis kahalalidefaciens*	Maintenance	[73]
Alteramides	3	octocoral	*Pseudoalteromonas sp.*	Maintenance	[74]
Stilbenenes (3,5-Dihydroxy-4-Isopropyl-Trans-Stilbene, Stilbene Epoxide, Prolbenes A-B Proline Conjugated)	3	nematodes *Heterorhabditis* spp.	*Photorhabdus luminescens*	Maintenance	[75]
L-Proline	3	Nematodes and insect larvae	*Photorhabdus luminescens and Xenorhabdus spp.*	Establishment	[76]
Diaphorin	3	psyllid *Diaphorina citri*	*Candidatus Profftella armatura*	Maintenance	[77]
Nystatin P1	3	ant *Acromyrmex octospinosus*	*Pseudonocardia*	Maintenance	[78]
Candicidin Macrolides	3	*Acromyrmex octospinosus*	*Streptomyces sp.*	Maintenance	[79]
2,3,5-Trimethylpyrazine and 2,3,5,6-Tetramethylpyrazine	3	olive fruit fly *Bactrocera dorsalis*	*Bacillus sp.*	Establishment	[80]
Phenol	3	beetles *Costelytra zealandica*	*Morganella morganii*	Maintenance	[81]
(Lmw) Succinoglycan	3	*Medicago sativa* (alfalfa)	*Sinorhizobium meliloti*	Establishment	[82,83]
Luteolin	3	*Medicago sativa* (alfalfa)	*Sinorhizobium meliloti*	Establishment	[84]
Betaine	3	*Medicago sativa* (alfalfa)	*Sinorhizobium meliloti*	Maintenance	[85]
Lugdunin	3	human nasal microbiota	*Staphylococcus lugdunensis*	Maintenance	[86]

Molecule	Symbiosis score*	Host	Symbiont	Symbiosis involvement	Reference
Sphingolipids (E.G., Ceramide Phosphoethanolamine)	3	mammalian hosts (murine-human gut)	*Bacteroides thetaiotaomicron*	Maintenance	87
Lipooligosaccharide (Los)	3	mammalian hosts (murine-human gut)	*Bacteroides thetaiotaomicron*	Maintenance	88
Sterols (Cholesterol And Phytosterols)	3	*Waminoa* sp. acoel flatworms	*Amphidinium dinoflagellate algae*	Unclear	89
Attinimicin	3	*Attine ants (Acromyrmex, Apterostigma, Trachymyrmex)*	*Pseudonocardia spp.*	Maintenance	90
Burkholdine 1213	3	*Atta, Acromyrmex, Paratrachymyrmex, Mycetomoellerius, Sericomyrmex, Mycetophylax, Cyphomyrmex, Myrmicocrypta, Apterostigma*	*Burkholderia spp.*	Maintenance	91
Pyrrolnitrin	3	*Atta, Acromyrmex, Paratrachymyrmex, Mycetomoellerius, Sericomyrmex, Mycetophylax, Cyphomyrmex, Myrmicocrypta, Apterostigma*	*Burkholderia spp.*	Maintenance	91

***Symbiosis score 1.** Chemical structure has been confirmed; **Symbiosis score 2.** Both chemical structure and molecule's producer have been confirmed; **Symbiosis score 3.** All the following, chemical structure, molecule's producer and bioassay confirming their involvement in symbiosis have been provided.

References

(1) Gil-Turnes, M., Sofia; Hay, M. E.; Fenical, W. Symbiotic Marine Bacteria Chemically Defend Crustacean Embryos from a Pathogenic Fungus. *Science* **1989**, *246* (4926), 116–118. https://doi.org/10.1126/science.2781297.

(2) Robinson, S. J.; Tenney, K.; Yee, D. F.; Martinez, L.; Media, J. E.; Valeriote, F. A.; van Soest, R. W. M.; Crews, P. Probing the Bioactive Constituents from Chemotypes of the Sponge Psammocinia Aff. Bulbosa. *J. Nat. Prod.* **2007**, *70* (6), 1002–1009. https://doi.org/10.1021/np070171i.

(3) Meng, A.; Corre, E.; Probert, I.; Gutierrez-Rodriguez, A.; Siano, R.; Annamale, A.; Alberti, A.; Da Silva, C.; Wincker, P.; Le Crom, S.; Not, F.; Bittner, L. Analysis of the Genomic Basis of Functional Diversity in Dinoflagellates Using a Transcriptome-Based Sequence Similarity Network. *Mol. Ecol.* **2018**, *27* (10), 2365–2380. https://doi.org/10.1111/mec.14579.

(4) Deng, H.; Shang, X.; Zhu, H.; Huang, N.; Wang, L.; Sun, M. Saxitoxin: A Comprehensive Review of Its History, Structure, Toxicology, Biosynthesis, Detection, and Preventive Implications. *Mar. Drugs* **2025**, *23* (7), 277. https://doi.org/10.3390/md23070277.

(5) Cusick, K. D.; Sayler, G. S. An Overview on the Marine Neurotoxin, Saxitoxin: Genetics, Molecular Targets, Methods of Detection and Ecological Functions. *Mar. Drugs* **2013**, *11* (4), 991–1018. https://doi.org/10.3390/md11040991.

(6) Daly, J. W. Marine Toxins and Nonmarine Toxins: Convergence or Symbiotic Organisms? *J. Nat. Prod.* **2004**, *67* (8), 1211–1215. https://doi.org/10.1021/np040016t.

(7) Chiou, Y.-J.; Chan, Y.-F.; Yu, S.-P.; Lu, C.-Y.; Hsiao, S. S.-Y.; Chiang, P.-W.; Hsu, T.-C.; Liu, P.-Y.; Wada, N.; Lee, Y.; Jane, W.-N.; Lee, D.-C.; Huang, Y.-W.; Tang, S.-L. Similar but Different: Characterization of dddD Gene–Mediated DMSP Metabolism among Coral-Associated Endozoicomonas. *Sci. Adv.* **2023**, *9* (47), eadk1910. https://doi.org/10.1126/sciadv.adk1910.
(8) Dillon, R. J.; Vennard, C. T.; Charnley, A. K. Exploitation of Gut Bacteria in the Locust. *Nature* **2000**, *403* (6772), 851–851. https://doi.org/10.1038/35002669.
(9) Olofsson, T. C.; Butler, È.; Markowicz, P.; Lindholm, C.; Larsson, L.; Vásquez, A. Lactic Acid Bacterial Symbionts in Honeybees – an Unknown Key to Honey's Antimicrobial and Therapeutic Activities. *Int. Wound J.* **2016**, *13* (5), 668–679. https://doi.org/10.1111/iwj.12345.
(10) Scott, J. J.; Oh, D.-C.; Yuceer, M. C.; Klepzig, K. D.; Clardy, J.; Currie, C. R. Bacterial Protection of Beetle-Fungus Mutualism. *Science* **2008**, *322* (5898), 63–63. https://doi.org/10.1126/science.1160423.
(11) Savana da Silva, J.; Soares de Carvalho, T.; Valentim dos Santos, J.; de Almeida Ribeiro, P. R.; de Souza Moreira, F. M. Formononetin Stimulates Mycorrhizal Fungi Colonization on the Surface of Active Root Nodules in Soybean. *Symbiosis* **2017**, *71* (1), 27–34. https://doi.org/10.1007/s13199-016-0408-9.
(12) Partida-Martinez, L. P.; Groth, I.; Schmitt, I.; Richter, W.; Roth, M.; Hertweck, C. Burkholderia Rhizoxinica Sp. Nov. and Burkholderia Endofungorum Sp. Nov., Bacterial Endosymbionts of the Plant-Pathogenic Fungus Rhizopus Microsporus. *Int. J. Syst. Evol. Microbiol.* **2007**, *57* (11), 2583–2590. https://doi.org/10.1099/ijs.0.64660-0.
(13) Davidson, S. K.; Allen, S. W.; Lim, G. E.; Anderson, C. M.; Haygood, M. G. Evidence for the Biosynthesis of Bryostatins by the Bacterial Symbiont "Candidatus Endobugula Sertula" of the BryozoanBugula Neritina. *Appl. Environ. Microbiol.* **2001**, *67* (10), 4531–4537. https://doi.org/10.1128/AEM.67.10.4531-4537.2001.
(14) Hildebrand, M.; Waggoner, L. E.; Liu, H.; Sudek, S.; Allen, S.; Anderson, C.; Sherman, D. H.; Haygood, M. bryA: An Unusual Modular Polyketide Synthase Gene from the Uncultivated Bacterial Symbiont of the Marine Bryozoan Bugula Neritina. *Chem. Biol.* **2004**, *11* (11), 1543–1552. https://doi.org/10.1016/j.chembiol.2004.08.018.
(15) Sharp, K. H.; Davidson, S. K.; Haygood, M. G. Localization of 'Candidatus Endobugula Sertula' and the Bryostatins throughout the Life Cycle of the Bryozoan Bugula Neritina. *ISME J.* **2007**, *1* (8), 693–702. https://doi.org/10.1038/ismej.2007.78.
(16) Buchholz, T. J.; Rath, C. M.; Lopanik, N. B.; Gardner, N. P.; Håkansson, K.; Sherman, D. H. Polyketide β-Branching in Bryostatin Biosynthesis: Identification of Surrogate Acetyl-ACP Donors for BryR, an HMG-ACP Synthase. *Chem. Biol.* **2010**, *17* (10), 1092–1100. https://doi.org/10.1016/j.chembiol.2010.08.008.
(17) Ridley, C. P.; Bergquist, P. R.; Harper, M. K.; Faulkner, D. J.; Hooper, J. N. A.; Haygood, M. G. Speciation and Biosynthetic Variation in Four Dictyoceratid Sponges and Their Cyanobacterial Symbiont, Oscillatoria Spongeliae. *Chem. Biol.* **2005**, *12* (3), 397–406. https://doi.org/10.1016/j.chembiol.2005.02.003.

(18) MacMillan, J. B.; Trousdale, E. K.; Molinski, T. F. Structure of (−)-Neodysidenin from Dysidea Herbacea. Implications for Biosynthesis of 5,5,5-Trichloroleucine Peptides. *Org. Lett.* **2000**, *2* (17), 2721–2723. https://doi.org/10.1021/ol006326u.
(19) Piel, J.; Hui, D.; Wen, G.; Butzke, D.; Platzer, M.; Fusetani, N.; Matsunaga, S. Antitumor Polyketide Biosynthesis by an Uncultivated Bacterial Symbiont of the Marine Sponge Theonella Swinhoei. *Proc. Natl. Acad. Sci.* **2004**, *101* (46), 16222–16227. https://doi.org/10.1073/pnas.0405976101.
(20) Freeman, M. F.; Helf, M. J.; Bhushan, A.; Morinaka, B. I.; Piel, J. Seven Enzymes Create Extraordinary Molecular Complexity in an Uncultivated Bacterium. *Nat. Chem.* **2017**, *9* (4), 387–395. https://doi.org/10.1038/nchem.2666.
(21) Gromek, S. M.; Suria, A. M.; Fullmer, M. S.; Garcia, J. L.; Gogarten, J. P.; Nyholm, S. V.; Balunas, M. J. Leisingera Sp. JC1, a Bacterial Isolate from Hawaiian Bobtail Squid Eggs, Produces Indigoidine and Differentially Inhibits Vibrios. *Front. Microbiol.* **2016**, *7*. https://doi.org/10.3389/fmicb.2016.01342.
(22) Suria, A. M.; Tan, K. C.; Kerwin, A. H.; Gitzel, L.; Abini-Agbomson, L.; Bertenshaw, J. M.; Sewell, J.; Nyholm, S. V.; Balunas, M. J. Hawaiian Bobtail Squid Symbionts Inhibit Marine Bacteria via Production of Specialized Metabolites, Including New Bromoalterochromides BAC-D/D′. *mSphere* **2020**, *5* (4), 10.1128/msphere.00166-20. https://doi.org/10.1128/msphere.00166-20.
(23) Braña, A. F.; Fiedler, H.-P.; Nava, H.; González, V.; Sarmiento-Vizcaíno, A.; Molina, A.; Acuña, J. L.; García, L. A.; Blanco, G. Two Streptomyces Species Producing Antibiotic, Antitumor, and Anti-Inflammatory Compounds Are Widespread Among Intertidal Macroalgae and Deep-Sea Coral Reef Invertebrates from the Central Cantabrian Sea. *Microb. Ecol.* **2015**, *69* (3), 512–524. https://doi.org/10.1007/s00248-014-0508-0.
(24) Wang, W.; Ji, J.; Li, X.; Wang, J.; Li, S.; Pan, G.; Fan, K.; Yang, K. Angucyclines as Signals Modulate the Behaviors of Streptomyces Coelicolor. *Proc. Natl. Acad. Sci.* **2014**, *111* (15), 5688–5693. https://doi.org/10.1073/pnas.1324253111.
(25) Rao, M.; Smith, B. C.; Marletta, M. A. Nitric Oxide Mediates Biofilm Formation and Symbiosis in Silicibacter Sp. Strain TrichCH4B. *mBio* **2015**, *6* (3), 10.1128/mbio.00206-15. https://doi.org/10.1128/mbio.00206-15.
(26) Davidson, S. K.; Koropatnick, T. A.; Kossmehl, R.; Sycuro, L.; McFall-Ngai, M. J. NO Means ‘Yes’ in the Squid-Vibrio Symbiosis: Nitric Oxide (NO) during the Initial Stages of a Beneficial Association. *Cell. Microbiol.* **2004**, *6* (12), 1139–1151. https://doi.org/10.1111/j.1462-5822.2004.00429.x.
(27) Matsuo, Y.; Imagawa, H.; Nishizawa, M.; Shizuri, Y. Isolation of an Algal Morphogenesis Inducer from a Marine Bacterium. *Science* **2005**, *307* (5715), 1598–1598. https://doi.org/10.1126/science.1105486.
(28) Schmidt, E. W.; Donia, M. S. Life in Cellulose Houses: Symbiotic Bacterial Biosynthesis of Ascidian Drugs and Drug Leads. *Curr. Opin. Biotechnol.* **2010**, *21* (6), 827–833. https://doi.org/10.1016/j.copbio.2010.10.006.
(29) Park, H. B.; Perez, C. E.; Perry, E. K.; Crawford, J. M. Activating and Attenuating the Amicoumacin Antibiotics. *Molecules* **2016**, *21* (7), 824. https://doi.org/10.3390/molecules21070824.
(30) Singh, S.; Orr, D.; Divinagracia, E.; McGraw, J.; Dorff, K.; Forst, S. Role of Secondary Metabolites in Establishment of the Mutualistic Partnership between

Xenorhabdus Nematophila and the Entomopathogenic Nematode Steinernema Carpocapsae. *Appl. Environ. Microbiol.* **2015**, *81* (2), 754–764. https://doi.org/10.1128/AEM.02650-14.
(31) Imai, Y.; Meyer, K. J.; Iinishi, A.; Favre-Godal, Q.; Green, R.; Manuse, S.; Caboni, M.; Mori, M.; Niles, S.; Ghiglieri, M.; Honrao, C.; Ma, X.; Guo, J. J.; Makriyannis, A.; Linares-Otoya, L.; Böhringer, N.; Wuisan, Z. G.; Kaur, H.; Wu, R.; Mateus, A.; Typas, A.; Savitski, M. M.; Espinoza, J. L.; O'Rourke, A.; Nelson, K. E.; Hiller, S.; Noinaj, N.; Schäberle, T. F.; D'Onofrio, A.; Lewis, K. A New Antibiotic Selectively Kills Gram-Negative Pathogens. *Nature* **2019**, *576* (7787), 459–464. https://doi.org/10.1038/s41586-019-1791-1.
(32) Crawford, J. M.; Kontnik, R.; Clardy, J. Regulating Alternative Lifestyles in Entomopathogenic Bacteria. *Curr. Biol.* **2010**, *20* (1), 69–74. https://doi.org/10.1016/j.cub.2009.10.059.
(33) Piel, J.; Butzke, D.; Fusetani, N.; Hui, D.; Platzer, M.; Wen, G.; Matsunaga, S. Exploring the Chemistry of Uncultivated Bacterial Symbionts: Antitumor Polyketides of the Pederin Family. *J. Nat. Prod.* **2005**, *68* (3), 472–479. https://doi.org/10.1021/np049612d.
(34) Oh, D.-C.; Poulsen, M.; Currie, C. R.; Clardy, J. Dentigerumycin: A Bacterial Mediator of an Ant-Fungus Symbiosis. *Nat. Chem. Biol.* **2009**, *5* (6), 391–393. https://doi.org/10.1038/nchembio.159.
(35) Sit, C. S.; Ruzzini, A. C.; Van Arnam, E. B.; Ramadhar, T. R.; Currie, C. R.; Clardy, J. Variable Genetic Architectures Produce Virtually Identical Molecules in Bacterial Symbionts of Fungus-Growing Ants. *Proc. Natl. Acad. Sci.* **2015**, *112* (43), 13150–13154. https://doi.org/10.1073/pnas.1515348112.
(36) Van Arnam, E. B.; Ruzzini, A. C.; Sit, C. S.; Horn, H.; Pinto-Tomás, A. A.; Currie, C. R.; Clardy, J. Selvamicin, an Atypical Antifungal Polyene from Two Alternative Genomic Contexts. *Proc. Natl. Acad. Sci.* **2016**, *113* (46), 12940–12945. https://doi.org/10.1073/pnas.1613285113.
(37) Van Arnam, E. B.; Ruzzini, A. C.; Sit, C. S.; Currie, C. R.; Clardy, J. A Rebeccamycin Analog Provides Plasmid-Encoded Niche Defense. *J. Am. Chem. Soc.* **2015**, *137* (45), 14272–14274. https://doi.org/10.1021/jacs.5b09794.
(38) Seipke, R. F.; Barke, J.; Brearley, C.; Hill, L.; Yu, D. W.; Goss, R. J. M.; Hutchings, M. I. A Single Streptomyces Symbiont Makes Multiple Antifungals to Support the Fungus Farming Ant Acromyrmex Octospinosus. *PLOS ONE* **2011**, *6* (8), e22028. https://doi.org/10.1371/journal.pone.0022028.
(39) Um, S.; Bach, D.-H.; Shin, B.; Ahn, C.-H.; Kim, S.-H.; Bang, H.-S.; Oh, K.-B.; Lee, S. K.; Shin, J.; Oh, D.-C. Naphthoquinone–Oxindole Alkaloids, Coprisidins A and B, from a Gut-Associated Bacterium in the Dung Beetle, Copris Tripartitus. *Org. Lett.* **2016**, *18* (22), 5792–5795. https://doi.org/10.1021/acs.orglett.6b02555.
(40) Kaltenpoth, M.; Goettler, W.; Dale, C.; Stubblefield, J. W.; Herzner, G.; Roeser-Mueller, K.; Strohm, E. 'Candidatus Streptomyces Philanthi', an Endosymbiotic Streptomycete in the Antennae of Philanthus Digger Wasps. *Int. J. Syst. Evol. Microbiol.* **2006**, *56* (6), 1403–1411. https://doi.org/10.1099/ijs.0.64117-0.
(41) Kroiss, J.; Kaltenpoth, M.; Schneider, B.; Schwinger, M.-G.; Hertweck, C.; Maddula, R. K.; Strohm, E.; Svatoš, A. Symbiotic Streptomycetes Provide Antibiotic

Combination Prophylaxis for Wasp Offspring. *Nat. Chem. Biol.* **2010**, *6* (4), 261–263. https://doi.org/10.1038/nchembio.331.
(42) Kaltenpoth, M.; Yildirim, E.; Gürbüz, M. F.; Herzner, G.; Strohm, E. Refining the Roots of the Beewolf-Streptomyces Symbiosis: Antennal Symbionts in the Rare Genus Philanthinus (Hymenoptera, Crabronidae). *Appl. Environ. Microbiol.* **2012**, *78* (3), 822–827. https://doi.org/10.1128/AEM.06809-11.
(43) Adnani, N.; Rajski, S. R.; Bugni, T. S. Symbiosis-Inspired Approaches to Antibiotic Discovery. *Nat. Prod. Rep.* **2017**, *34* (7), 784–814. https://doi.org/10.1039/c7np00009j.
(44) Engel, P.; Vizcaino, M. I.; Crawford, J. M. Gut Symbionts from Distinct Hosts Exhibit Genotoxic Activity via Divergent Colibactin Biosynthesis Pathways. *Appl. Environ. Microbiol.* **2015**, *81* (4), 1502–1512. https://doi.org/10.1128/AEM.03283-14.
(45) Han, F.; He, X.; Chen, W.; Gai, H.; Bai, X.; He, Y.; Takeshima, K.; Ohwada, T.; Wei, M.; Xie, F. Involvement of a Novel TetR-Like Regulator (BdtR) of Bradyrhizobium Diazoefficiens in the Efflux of Isoflavonoid Genistein. *Mol. Plant-Microbe Interactions®* **2020**, *33* (12), 1411–1423. https://doi.org/10.1094/MPMI-08-20-0243-R.
(46) Yuhashi, K.-I.; Ichikawa, N.; Ezura, H.; Akao, S.; Minakawa, Y.; Nukui, N.; Yasuta, T.; Minamisawa, K. Rhizobitoxine Production by Bradyrhizobium Elkanii Enhances Nodulation and Competitiveness onMacroptilium Atropurpureum. *Appl. Environ. Microbiol.* **2000**, *66* (6), 2658–2663. https://doi.org/10.1128/AEM.66.6.2658-2663.2000.
(47) Hu, L.; Robert, C. A. M.; Cadot, S.; Zhang, X.; Ye, M.; Li, B.; Manzo, D.; Chervet, N.; Steinger, T.; van der Heijden, M. G. A.; Schlaeppi, K.; Erb, M. Root Exudate Metabolites Drive Plant-Soil Feedbacks on Growth and Defense by Shaping the Rhizosphere Microbiota. *Nat. Commun.* **2018**, *9* (1), 2738. https://doi.org/10.1038/s41467-018-05122-7.
(48) Subramanian, S.; Stacey, G.; Yu, O. Endogenous Isoflavones Are Essential for the Establishment of Symbiosis between Soybean and Bradyrhizobium Japonicum. *Plant J.* **2006**, *48* (2), 261–273. https://doi.org/10.1111/j.1365-313X.2006.02874.x.
(49) Phillips, D. A.; Joseph, C. M.; Maxwell, C. A. Trigonelline and Stachydrine Released from Alfalfa Seeds Activate NodD2 Protein in Rhizobium Meliloti 1. *Plant Physiol.* **1992**, *99* (4), 1526–1531. https://doi.org/10.1104/pp.99.4.1526.
(50) Guo, D.; Li, J.; Liu, P.; Wang, Y.; Cao, N.; Fang, X.; Wang, T.; Dong, J. The Jasmonate Pathway Promotes Nodule Symbiosis and Suppresses Host Plant Defense in Medicago Truncatula. *Mol. Plant* **2024**, *17* (8), 1183–1203. https://doi.org/10.1016/j.molp.2024.06.004.
(51) Chen, S.; Chen, D.; Cai, R.; Cui, H.; Long, Y.; Lu, Y.; Li, C.; She, Z. Cytotoxic and Antibacterial Preussomerins from the Mangrove Endophytic Fungus Lasiodiplodia Theobromae ZJ-HQ1. *J. Nat. Prod.* **2016**, *79* (9), 2397–2402. https://doi.org/10.1021/acs.jnatprod.6b00639.
(52) Barnes, E. C.; Jumpathong, J.; Lumyong, S.; Voigt, K.; Hertweck, C. Daldionin, an Unprecedented Binaphthyl Derivative, and Diverse Polyketide Congeners from a Fungal Orchid Endophyte. *Chem. – Eur. J.* **2016**, *22* (13), 4551–4555. https://doi.org/10.1002/chem.201504005.

(53) Gehringer, M. M.; Adler, L.; Roberts, A. A.; Moffitt, M. C.; Mihali, T. K.; Mills, T. J. T.; Fieker, C.; Neilan, B. A. Nodularin, a Cyanobacterial Toxin, Is Synthesized in Planta by Symbiotic Nostoc Sp. *ISME J.* **2012**, *6* (10), 1834–1847. https://doi.org/10.1038/ismej.2012.25.
(54) Cox, P. A.; Banack, S. A.; Murch, S. J.; Rasmussen, U.; Tien, G.; Bidigare, R. R.; Metcalf, J. S.; Morrison, L. F.; Codd, G. A.; Bergman, B. Diverse Taxa of Cyanobacteria Produce β-N-Methylamino-l-Alanine, a Neurotoxic Amino Acid. *Proc. Natl. Acad. Sci.* **2005**, *102* (14), 5074–5078. https://doi.org/10.1073/pnas.0501526102.
(55) Gamba, A. G.; Oakley, C. A.; Ashley, I. A.; Grossman, A. R.; Weis, V. M.; Suggett, D. J.; Davy, S. K. Oxylipin Receptors and Their Role in Inter-Partner Signalling in a Model Cnidarian-Dinoflagellate Symbiosis. *Environ. Microbiol.* **2024**, *26* (12), e70015. https://doi.org/10.1111/1462-2920.70015.
(56) Ruiz-Rodríguez, M.; Martínez-Bueno, M.; Martín-Vivaldi, M.; Valdivia, E.; Soler, J. J. Bacteriocins with a Broader Antimicrobial Spectrum Prevail in Enterococcal Symbionts Isolated from the Hoopoe’s Uropygial Gland. *FEMS Microbiol. Ecol.* **2013**, *85* (3), 495–502. https://doi.org/10.1111/1574-6941.12138.
(57) Soler, J. J.; Barón, M. D.; Martínez-Renau, E.; Zhang, L.; Liang, W.; Martín-Vivaldi, M. Nesting Hoopoes Cultivate in Their Uropygial Gland the Microbial Symbionts with the Highest Antimicrobial Capacity. *Sci. Rep.* **2024**, *14* (1), 30797. https://doi.org/10.1038/s41598-024-81062-1.
(58) Donia, M. S.; Cimermancic, P.; Schulze, C. J.; Wieland Brown, L. C.; Martin, J.; Mitreva, M.; Clardy, J.; Linington, R. G.; Fischbach, M. A. A Systematic Analysis of Biosynthetic Gene Clusters in the Human Microbiome Reveals a Common Family of Antibiotics. *Cell* **2014**, *158* (6), 1402–1414. https://doi.org/10.1016/j.cell.2014.08.032.
(59) Chu, J.; Vila-Farres, X.; Inoyama, D.; Ternei, M.; Cohen, L. J.; Gordon, E. A.; Reddy, B. V. B.; Charlop-Powers, Z.; Zebroski, H. A.; Gallardo-Macias, R.; Jaskowski, M.; Satish, S.; Park, S.; Perlin, D. S.; Freundlich, J. S.; Brady, S. F. Discovery of MRSA Active Antibiotics Using Primary Sequence from the Human Microbiome. *Nat. Chem. Biol.* **2016**, *12* (12), 1004–1006. https://doi.org/10.1038/nchembio.2207.
(60) Healy, A. R.; Vizcaino, M. I.; Crawford, J. M.; Herzon, S. B. Convergent and Modular Synthesis of Candidate Precolibactins. Structural Revision of Precolibactin A. *J. Am. Chem. Soc.* **2016**, *138* (16), 5426–5432. https://doi.org/10.1021/jacs.6b02276.
(61) Chang, W.; Zhang, M.; Li, Y.; Li, X.; Gao, Y.; Xie, Z.; Lou, H. Lichen Endophyte Derived Pyridoxatin Inactivates *Candida* Growth by Interfering with Ergosterol Biosynthesis. *Biochim. Biophys. Acta BBA - Gen. Subj.* **2015**, *1850* (9), 1762–1771. https://doi.org/10.1016/j.bbagen.2015.05.005.
(62) Kaasalainen, U.; Fewer, D. P.; Jokela, J.; Wahlsten, M.; Sivonen, K.; Rikkinen, J. Cyanobacteria Produce a High Variety of Hepatotoxic Peptides in Lichen Symbiosis. *Proc. Natl. Acad. Sci.* **2012**, *109* (15), 5886–5891. https://doi.org/10.1073/pnas.1200279109.
(63) Kampa, A.; Gagunashvili, A. N.; Gulder, T. A. M.; Morinaka, B. I.; Daolio, C.; Godejohann, M.; Miao, V. P. W.; Piel, J.; Andrésson, Ó. S. Metagenomic Natural

Product Discovery in Lichen Provides Evidence for a Family of Biosynthetic Pathways in Diverse Symbioses. *Proc. Natl. Acad. Sci.* **2013**, *110* (33), E3129–E3137. https://doi.org/10.1073/pnas.1305867110.
(64) Davies, J.; Wang, H.; Taylor, T.; Warabi, K.; Huang, X.-H.; Andersen, R. J. Uncialamycin, A New Enediyne Antibiotic. *Org. Lett.* **2005**, *7* (23), 5233–5236. https://doi.org/10.1021/ol052081f.
(65) Cheenpracha, S.; Vidor, N. B.; Yoshida, W. Y.; Davies, J.; Chang, L. C. Coumabiocins A−F, Aminocoumarins from an Organic Extract of Streptomyces Sp. L-4-4. *J. Nat. Prod.* **2010**, *73* (5), 880–884. https://doi.org/10.1021/np900843b.
(66) Schoenian, I.; Spiteller, M.; Ghaste, M.; Wirth, R.; Herz, H.; Spiteller, D. Chemical Basis of the Synergism and Antagonism in Microbial Communities in the Nests of Leaf-Cutting Ants. *Proc. Natl. Acad. Sci.* **2011**, *108* (5), 1955–1960. https://doi.org/10.1073/pnas.1008441108.
(67) Carr, G.; Derbyshire, E. R.; Caldera, E.; Currie, C. R.; Clardy, J. Antibiotic and Antimalarial Quinones from Fungus-Growing Ant-Associated Pseudonocardia Sp. *J. Nat. Prod.* **2012**, *75* (10), 1806–1809. https://doi.org/10.1021/np300380t.
(68) Zink, K. E.; Ludvik, D. A.; Lazzara, P. R.; Moore, T. W.; Mandel, M. J.; Sanchez, L. M. A Small Molecule Coordinates Symbiotic Behaviors in a Host Organ. *mBio* **2021**, *12* (2), 10.1128/mbio.03637-20. https://doi.org/10.1128/mbio.03637-20.
(69) Seyedsayamdost, M. R.; Carr, G.; Kolter, R.; Clardy, J. Roseobacticides: Small Molecule Modulators of an Algal-Bacterial Symbiosis. *J. Am. Chem. Soc.* **2011**, *133* (45), 18343–18349. https://doi.org/10.1021/ja207172s.
(70) Seyedsayamdost, M. R.; Case, R. J.; Kolter, R.; Clardy, J. The Jekyll-and-Hyde Chemistry of Phaeobacter Gallaeciensis. *Nat. Chem.* **2011**, *3* (4), 331–335. https://doi.org/10.1038/nchem.1002.
(71) Schmidt, E. W.; Nelson, J. T.; Rasko, D. A.; Sudek, S.; Eisen, J. A.; Haygood, M. G.; Ravel, J. Patellamide A and C Biosynthesis by a Microcin-like Pathway in Prochloron Didemni, the Cyanobacterial Symbiont of Lissoclinum Patella. *Proc. Natl. Acad. Sci.* **2005**, *102* (20), 7315–7320. https://doi.org/10.1073/pnas.0501424102.
(72) Donia, M. S.; Hathaway, B. J.; Sudek, S.; Haygood, M. G.; Rosovitz, M. J.; Ravel, J.; Schmidt, E. W. Natural Combinatorial Peptide Libraries in Cyanobacterial Symbionts of Marine Ascidians. *Nat. Chem. Biol.* **2006**, *2* (12), 729–735. https://doi.org/10.1038/nchembio829.
(73) Zan, J.; Li, Z.; Tianero, Ma. D.; Davis, J.; Hill, R. T.; Donia, M. S. A Microbial Factory for Defensive Kahalalides in a Tripartite Marine Symbiosis. *Science* **2019**, *364* (6445), eaaw6732. https://doi.org/10.1126/science.aaw6732.
(74) Moree, W. J.; McConnell, O. J.; Nguyen, D. D.; Sanchez, L. M.; Yang, Y.-L.; Zhao, X.; Liu, W.-T.; Boudreau, P. D.; Srinivasan, J.; Atencio, L.; Ballesteros, J.; Gavilán, R. G.; Torres-Mendoza, D.; Guzmán, H. M.; Gerwick, W. H.; Gutiérrez, M.; Dorrestein, P. C. Microbiota of Healthy Corals Are Active against Fungi in a Light-Dependent Manner. *ACS Chem. Biol.* **2014**, *9* (10), 2300–2308. https://doi.org/10.1021/cb500432j.
(75) Park, H. B.; Sampathkumar, P.; Perez, C. E.; Lee, J. H.; Tran, J.; Bonanno, J. B.; Hallem, E. A.; Almo, S. C.; Crawford, J. M. Stilbene Epoxidation and Detoxification in a Photorhabdus Luminescens-Nematode Symbiosis. *J. Biol. Chem.* **2017**, *292* (16), 6680–6694. https://doi.org/10.1074/jbc.M116.762542.

(76) Crawford, J. M.; Kontnik, R.; Clardy, J. Regulating Alternative Lifestyles in Entomopathogenic Bacteria. *Curr. Biol.* **2010**, *20* (1), 69–74. https://doi.org/10.1016/j.cub.2009.10.059.
(77) Nakabachi, A.; Ueoka, R.; Oshima, K.; Teta, R.; Mangoni, A.; Gurgui, M.; Oldham, N. J.; van Echten-Deckert, G.; Okamura, K.; Yamamoto, K.; Inoue, H.; Ohkuma, M.; Hongoh, Y.; Miyagishima, S.; Hattori, M.; Piel, J.; Fukatsu, T. Defensive Bacteriome Symbiont with a Drastically Reduced Genome. *Curr. Biol.* **2013**, *23* (15), 1478–1484. https://doi.org/10.1016/j.cub.2013.06.027.
(78) Barke, J.; Seipke, R. F.; Grüschow, S.; Heavens, D.; Drou, N.; Bibb, M. J.; Goss, R. J.; Yu, D. W.; Hutchings, M. I. A Mixed Community of Actinomycetes Produce Multiple Antibiotics for the Fungus Farming Ant Acromyrmex Octospinosus. *BMC Biol.* **2010**, *8* (1), 109. https://doi.org/10.1186/1741-7007-8-109.
(79) Haeder, S.; Wirth, R.; Herz, H.; Spiteller, D. Candicidin-Producing Streptomyces Support Leaf-Cutting Ants to Protect Their Fungus Garden against the Pathogenic Fungus Escovopsis. *Proc. Natl. Acad. Sci.* **2009**, *106* (12), 4742–4746. https://doi.org/10.1073/pnas.0812082106.
(80) Ren, L.; Ma, Y.; Xie, M.; Lu, Y.; Cheng, D. Rectal Bacteria Produce Sex Pheromones in the Male Oriental Fruit Fly. *Curr. Biol.* **2021**, *31* (10), 2220-2226.e4. https://doi.org/10.1016/j.cub.2021.02.046.
(81) Marshall, D. G.; Jackson, T. A.; Unelius, C. R.; Wee, S. L.; Young, S. D.; Townsend, R. J.; Suckling, D. M. Morganella Morganii Bacteria Produces Phenol as the Sex Pheromone of the New Zealand Grass Grub from Tyrosine in the Colleterial Gland. *Sci. Nat.* **2016**, *103* (7), 59. https://doi.org/10.1007/s00114-016-1380-1.
(82) Arnold, M. F. F.; Penterman, J.; Shabab, M.; Chen, E. J.; Walker, G. C. Important Late-Stage Symbiotic Role of the Sinorhizobium Meliloti Exopolysaccharide Succinoglycan. *J. Bacteriol.* **2018**, *200* (13), 10.1128/jb.00665-17. https://doi.org/10.1128/jb.00665-17.
(83) Jones, K. M.; Sharopova, N.; Lohar, D. P.; Zhang, J. Q.; VandenBosch, K. A.; Walker, G. C. Differential Response of the Plant Medicago Truncatula to Its Symbiont Sinorhizobium Meliloti or an Exopolysaccharide-Deficient Mutant. *Proc. Natl. Acad. Sci.* **2008**, *105* (2), 704–709. https://doi.org/10.1073/pnas.0709338105.
(84) Peck, M. C.; Fisher, R. F.; Long, S. R. Diverse Flavonoids Stimulate NodD1 Binding to Nod Gene Promoters in Sinorhizobium Meliloti. *J. Bacteriol.* **2006**, *188* (15), 5417–5427. https://doi.org/10.1128/jb.00376-06.
(85) Mandon, K.; Østerås, M.; Boncompagni, E.; Trinchant, J. C.; Spennato, G.; Poggi, M. C.; Le Rudulier, D. The Sinorhizobium Meliloti Glycine Betaine Biosynthetic Genes (betICBA) Are Induced by Choline and Highly Expressed in Bacteroids. *Mol. Plant-Microbe Interactions®* **2003**, *16* (8), 709–719. https://doi.org/10.1094/MPMI.2003.16.8.709.
(86) Zipperer, A.; Konnerth, M. C.; Laux, C.; Berscheid, A.; Janek, D.; Weidenmaier, C.; Burian, M.; Schilling, N. A.; Slavetinsky, C.; Marschal, M.; Willmann, M.; Kalbacher, H.; Schittek, B.; Brötz-Oesterhelt, H.; Grond, S.; Peschel, A.; Krismer, B. Human Commensals Producing a Novel Antibiotic Impair Pathogen Colonization. *Nature* **2016**, *535* (7613), 511–516. https://doi.org/10.1038/nature18634.
(87) Brown, E. M.; Ke, X.; Hitchcock, D.; Jeanfavre, S.; Avila-Pacheco, J.; Nakata, T.; Arthur, T. D.; Fornelos, N.; Heim, C.; Franzosa, E. A.; Watson, N.; Huttenhower, C.;

Haiser, H. J.; Dillow, G.; Graham, D. B.; Finlay, B. B.; Kostic, A. D.; Porter, J. A.; Vlamakis, H.; Clish, C. B.; Xavier, R. J. Bacteroides-Derived Sphingolipids Are Critical for Maintaining Intestinal Homeostasis and Symbiosis. *Cell Host Microbe* **2019**, *25* (5), 668-680.e7. https://doi.org/10.1016/j.chom.2019.04.002.
(88) Jacobson, A. N.; Choudhury, B. P.; Fischbach, M. A. The Biosynthesis of Lipooligosaccharide from Bacteroides Thetaiotaomicron. *mBio* **2018**, *9* (2), 10.1128/mbio.02289-17. https://doi.org/10.1128/mbio.02289-17.
(89) Bien, T.; Hambleton, E. A.; Dreisewerd, K.; Soltwisch, J. Molecular Insights into Symbiosis—Mapping Sterols in a Marine Flatworm-Algae-System Using High Spatial Resolution MALDI-2-MS Imaging with Ion Mobility Separation. *Anal. Bioanal. Chem.* **2021**, *413* (10), 2767–2777. https://doi.org/10.1007/s00216-020-03070-0.
(90) Fukuda, T. T. H.; Helfrich, E. J. N.; Mevers, E.; Melo, W. G. P.; Van Arnam, E. B.; Andes, D. R.; Currie, C. R.; Pupo, M. T.; Clardy, J. Specialized Metabolites Reveal Evolutionary History and Geographic Dispersion of a Multilateral Symbiosis. *ACS Cent. Sci.* **2021**, *7* (2), 292–299. https://doi.org/10.1021/acscentsci.0c00978.
(91) Francoeur, C. B.; May, D. S.; Thairu, M. W.; Hoang, D. Q.; Panthofer, O.; Bugni, T. S.; Pupo, M. T.; Clardy, J.; Pinto-Tomás, A. A.; Currie, C. R. Burkholderia from Fungus Gardens of Fungus-Growing Ants Produces Antifungals That Inhibit the Specialized Parasite Escovopsis. *Appl. Environ. Microbiol.* **2021**, *87* (14), e00178-21. https://doi.org/10.1128/AEM.00178-21.
